# 18F-FDG PET/CT for predicting major pathological response to neoadjuvant therapy in non-small cell lung cancer: a meta-analysis

**DOI:** 10.3389/fonc.2026.1730878

**Published:** 2026-05-15

**Authors:** Yaoyu Wu, Boxiao Hu, Dazhi Liu, Hao Meng

**Affiliations:** Department of Thoracic Surgery, Northern Theater General Hospital, Shenyang, Liaoning, China

**Keywords:** 18F-FDG, MPR, neoadjuvant therapy, NSCLC, PET/CT, SUVmax

## Abstract

**Objective:**

To comprehensively evaluate the diagnostic utility of metabolic parameter changes on 18F-fluorodeoxyglucose positron emission tomography/computed tomography (18F-FDG PET/CT) for predicting pathological response after neoadjuvant therapy in patients with non-small cell lung cancer (NSCLC).

**Methods:**

The Cochrane Library, Web of Science, Embase, and PubMed were comprehensively retrieved up until October 2024 to identify diagnostic test accuracy studies evaluating the predictive efficacy of 18F-FDG PET/CT for postoperative pathological response in NSCLC patients following neoadjuvant therapy. Two independent reviewers screened studies, collected information, and evaluated the risk of bias. Statistical analysis was conducted via Meta-Disc 1.4 and Stata 17.0 software.

**Results:**

Fourteen eligible investigations encompassing 1,315 subjects were incorporated. The findings revealed that for predicting major pathological response (MPR) after neoadjuvant therapy, the metabolic parameter ΔSUVmax% on 18F-FDG PET/CT achieved an area under the summary receiver operating characteristic curve (sAUC) of 0.96 (95% CI: 0.94-0.97), with pooled sensitivity and specificity of 0.87 (0.73-0.94) and 0.93 (0.84-0.97), respectively. Similarly, the parameter SUVmax itself yielded an sAUC of 0.95 (0.93-0.96), with sensitivity of 0.80 (0.63-0.91) and specificity of 0.94 (0.87-0.97). Stratified analyses indicated that the neoadjuvant treatment regimen (single-modality vs. combination therapy), the cut-off value (<55% vs. ≥55%), sample size (<40 vs. ≥40), and the proportion of adenocarcinoma in the study sample (<40% vs. ≥40%) were potential sources of heterogeneity.

**Conclusion:**

Quantitative metabolic metrics derived from 18F-FDG PET/CT (ΔSUVmax% and SUVmax) demonstrate high diagnostic performance for pathological response after neoadjuvant therapy in NSCLC. These metrics may serve as noninvasive adjunct tools for response assessment, supporting individualized neoadjuvant strategies and facilitating clinical trial design in the perioperative immunotherapy era. However, given the limited number and quality of the available studies, these findings require confirmation through high-quality research.

**Systematic review registration:**

https://www.crd.york.ac.uk/prospero/, identifier CRD42024612056.

## 1 Introduction

Globally, non-small cell lung cancer (NSCLC) constitutes roughly 85% of lung cancer cases and represents a major contributor to cancer-related mortality (1, 2). According to the 2021 World Health Organization (WHO) classification of thoracic tumors, the principal histological subtypes encompass adenocarcinoma (40%-50%), squamous cell carcinoma (20%-30%), and large cell carcinoma (10%-15%). Notably, about 30% of NSCLC individuals are diagnosed at a locally advanced stage (3, 4).

The evolution of neoadjuvant therapy has markedly transformed the clinical management of locally advanced NSCLC. Progression from traditional neoadjuvant chemotherapy (platinum-based doublet regimens) to emerging combination therapies involving immune checkpoint inhibitors has enabled not only increased R0 resection rates but also induction of significant pathological responses in some patients by reducing primary tumor T-stage and eliminating circulating micro-metastases (5–9). Major pathological response (MPR), characterized by ≤10% viable tumor cells, serves as the gold standard for evaluating neoadjuvant treatment efficacy and is endorsed by the International Association for the Study of Lung Cancer (IASLC) as a surrogate endpoint in clinical trials (10–12). Retrospective analyses indicate that patients achieving MPR experience a nearly twofold extension in median progression-free survival (PFS).

Noninvasive preoperative evaluation of therapeutic response is essential for optimizing individualized patient management. 18F-fluorodeoxyglucose positron emission tomography/computed tomography (18F-FDG PET/CT) constitutes a functional imaging technique that evaluates metabolic alterations within tumors, thereby reflecting biological activity and therapeutic response (13–15). A key clinical challenge involves leveraging imaging techniques to accurately predict MPR preoperatively for guiding treatment decisions. Although 18F-FDG PET/CT, by reflecting tumor glucose metabolic status (the Warburg effect), is routinely employed for NSCLC staging and response monitoring (16–20), its value in predicting MPR remains contentious. Existing evidence demonstrates significant heterogeneity in the predictive performance of metabolic parameters, such as SUVmax (median baseline range: 6.2-18.4) and ΔSUVmax (cut-off values varying between 40%-70%) (21–34). This discrepancy may stem from variations in PET acquisition protocols, inconsistent MPR assessment standards (particularly regarding lymph node evaluation), and differential metabolic response patterns induced by distinct treatment regimens. Consequently, the predictive accuracy and clinical utility of 18F-FDG PET/CT in this context require further elucidation.

Previous narrative and systematic reviews have preliminarily assessed the value of 18F-FDG PET/CT for evaluating responses to treatment in certain lung cancer subtypes (35), but they did not apply rigorous data screening or stratification of the included studies. Due to their earlier publication dates, these reviews did not incorporate evidence related to neoadjuvant immunotherapy. To address this gap, the current meta-analysis implemented strict data screening and classification using a harmonized definition of MPR. The analysis synthesized evidence from chemotherapy- and chemoimmunotherapy-based regimens and used meta-regression to explore between-study heterogeneity attributable to treatment regimen, histology, sample size, and ΔSUVmax% cutoffs.

## 2 Materials and methods

Our meta-analysis was implemented and reported per the Preferred Reporting Items for Systematic Reviews and Meta-Analyses (PRISMA) guidelines (36) and was prospectively registered with the International Prospective Register of Systematic Reviews (PROSPERO) database (CRD42024612056) in November 2024.

### 2.1 Literature retrieval

Comprehensive searches were performed in the Cochrane Library, Embase, Web of Science, and PubMed up until October 2024 to identify clinical diagnostic trials evaluating 18F-FDG PET/CT for assessing pathological response after neoadjuvant therapy in NSCLC subjects. The reference sections of all included studies were also scrutinized to ensure thorough retrieval. The methodology comprised both controlled vocabulary terms and free-text keywords, including but not limited to: bronchial non-small cell cancer, bronchial non-small cell carcinoma, lung non-small cell cancer, lung non-small cell carcinoma, Non-Small Cell Lung Cancer, neo adjuvant therapy, neo adjuvant treatment, Neoadjuvant Chemoradiation, Neoadjuvant Chemoradiation Therapy, Neoadjuvant Chemoradiation Treatment, Neoadjuvant Chemoradiotherapy, 18F FDG, 18F Fluorodeoxyglucose, 2 deoxy 2 fluoro d glucose f 18, Fluorodeoxyglucose F18, PET CT, PET CT Scan*, PET Imaging*, positron emission tomographic scan, positron emission tomographic scanning, Positron Emission Tomography, Positron Emission Tomography Imaging*. **Supplementary Table 1** illustrates the specifics.

### 2.2 Eligibility criteria

Inclusion criteria incorporated: (i) Study design: Diagnostic trials (prospective or retrospective).

(ii) Participants: Pathologically confirmed NSCLC subjects who received neoadjuvant therapy (radiotherapy/chemotherapy/immunotherapy/targeted therapy).
(iii) Diagnostic method: The index test was 18F-FDG PET/CT for evaluating pathological response post-neoadjuvant therapy, with the reference standard being postoperative pathological assessment.
(iv) Outcome measures: Availability of direct or calculable data for a 2×2 contingency table (false positive, true negative, false negative, true positive).

Exclusion criteria comprised: (i) Non-English articles; (ii) Animal experiments; (iii) Duplicate publications; (iv) Trials from which original data could not be extracted; (v) Investigations whose primary objective was unrelated to imaging predictive efficacy (e.g., solely assessing survival outcomes).

### 2.3 Literature screening and information collection

Two independent reviewers screened literature against the eligibility criteria. Subsequently, relevant information was collected from the eligible literature using a pre-defined form, followed by cross-verification between reviewers. Discrepancies were addressed through deliberation or arbitration by a third reviewer. Missing information was sought by contacting the authors whenever possible. Collected information included the first author, study design, publication year, age, sample size, geographical region, and diagnostic test results.

### 2.4 Risk of bias evaluation

The methodological quality of the eligible studies was appraised by two independent reviewers employing the Quality Assessment of Diagnostic Accuracy Studies-2 (QUADAS-2) tool, which is recommended by the Cochrane Collaboration and the National Institute for Health and Care Excellence (NICE) for assessing the diagnostic test accuracy (DTA). This tool evaluates DTA from four domains: patient selection, index test, reference standard, and flow and timing. The ROB was judged as ‘High’, ‘Low’, or ‘Unclear’. If all questions in a domain receive affirmative responses, the ROB is low. If any answer is ‘No’, the ROB is possible. If all answers are ‘No’, the ROB is high. A judgment of ‘Unclear’ indicates insufficient reporting detail to permit a clear assessment, suggesting potential ROB. Discrepancies were settled by consensus or third-party adjudication.

### 2.5 Statistical analysis

Statistical synthesis was implemented employing Stata 17.0, RevMan 5.3, and Meta-Disc 1.4 software. The Spearman correlation coefficient was computed to detect threshold effects. Heterogeneity arising from non-threshold effects was investigated utilizing the I² statistic, with I²>50% or P<0.10 suggesting substantial heterogeneity. Meta-regression and subgroup analyses explored possible origins of heterogeneity. Pooled estimates for specificity, positive likelihood ratio (PLR), sensitivity, negative likelihood ratio (NLR), and diagnostic odds ratio (DOR), as well as their 95% confidence intervals (CIs), were derived via random-effects models. Summary receiver operating characteristic (SROC) curves were generated, and the area under the curve (AUC) was computed to gauge overall diagnostic efficacy, where an AUC closer to one denotes higher accuracy. Sensitivity analysis employed the leave-one-out method. Publication bias was evaluated via Deeks’ funnel plots. The clinical utility of 18F-FDG PET/CT was illustrated using Fagan nomograms.

## 3 Results

### 3.1 Literature screening and study characteristics

**Figure 1:**
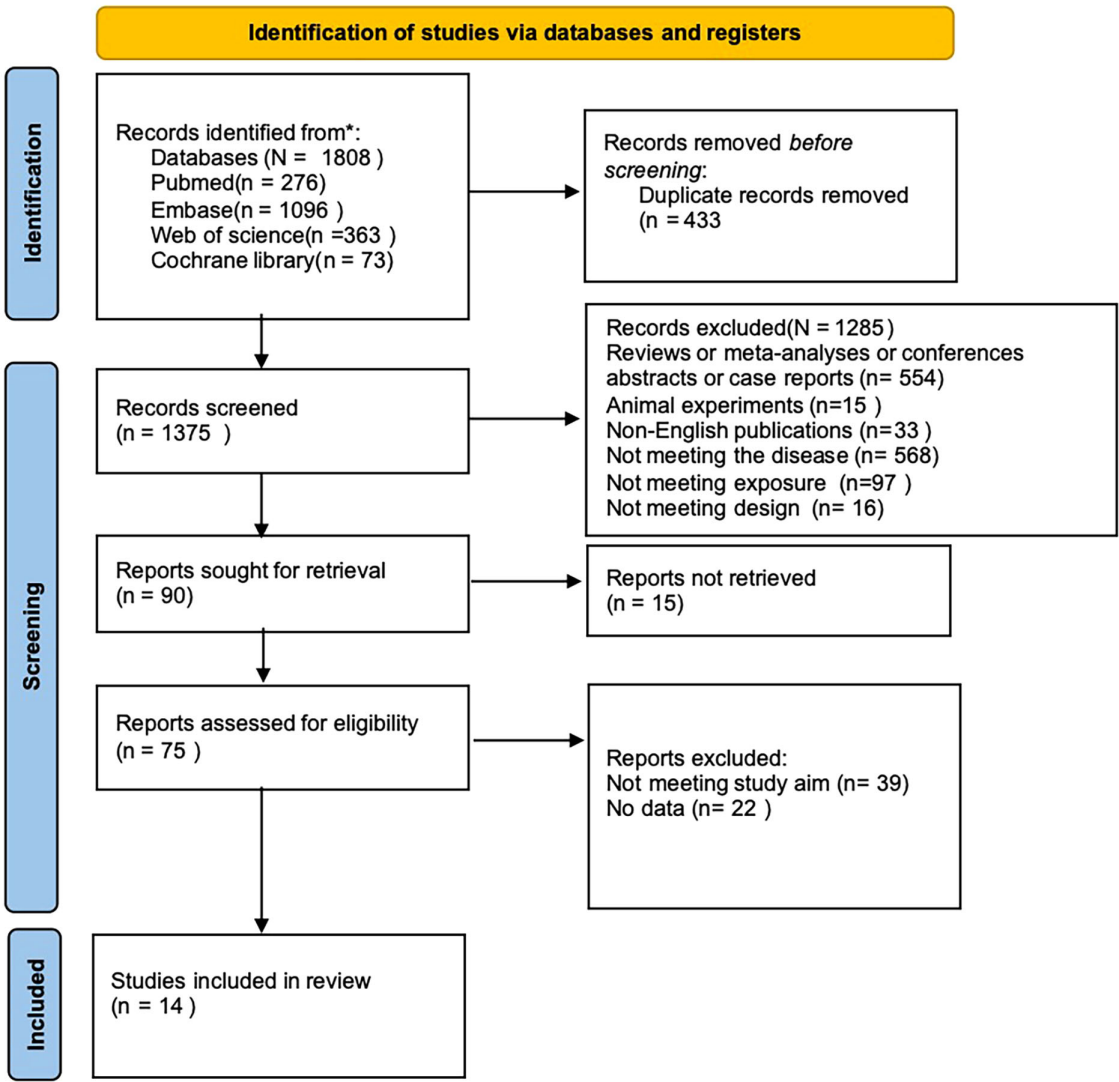
PRISMA flow diagram showing the study identification, screening, eligibility assessment, and inclusion process.

A preliminary retrieval yielded 1,808 articles, incorporating 276 from PubMed, 73 from the Cochrane Library, 1,096 from Embase, and 363 from Web of Science. After removing 483 duplicates, 1,285 articles were excluded based on abstract screening (reasons: animal experiments, non-English articles, reviews, irrelevant disease focus, or exposure). Full texts were acquired for the remaining 90 citations, of which 15 were inaccessible. Following a detailed assessment of the 75 remaining articles, 39 were deleted for not aligning with the research objective, and 22 for lacking relevant data. Ultimately, 14 articles reporting PET-CT data were incorporated (21–34). **Figure 1** presents the literature screening process.

The 14 eligible studies comprised 1,315 pathologically confirmed NSCLC patients (1,035 males, 280 females). Twelve studies (85.7%) employed a retrospective design, and ten (71.4%) were conducted in Asian populations. Four studies were published before 2020. Demographic and baseline clinical features of the subjects are detailed in **Supplementary Table 2**.

### 3.2 ROB assessment

**Figure 2:**
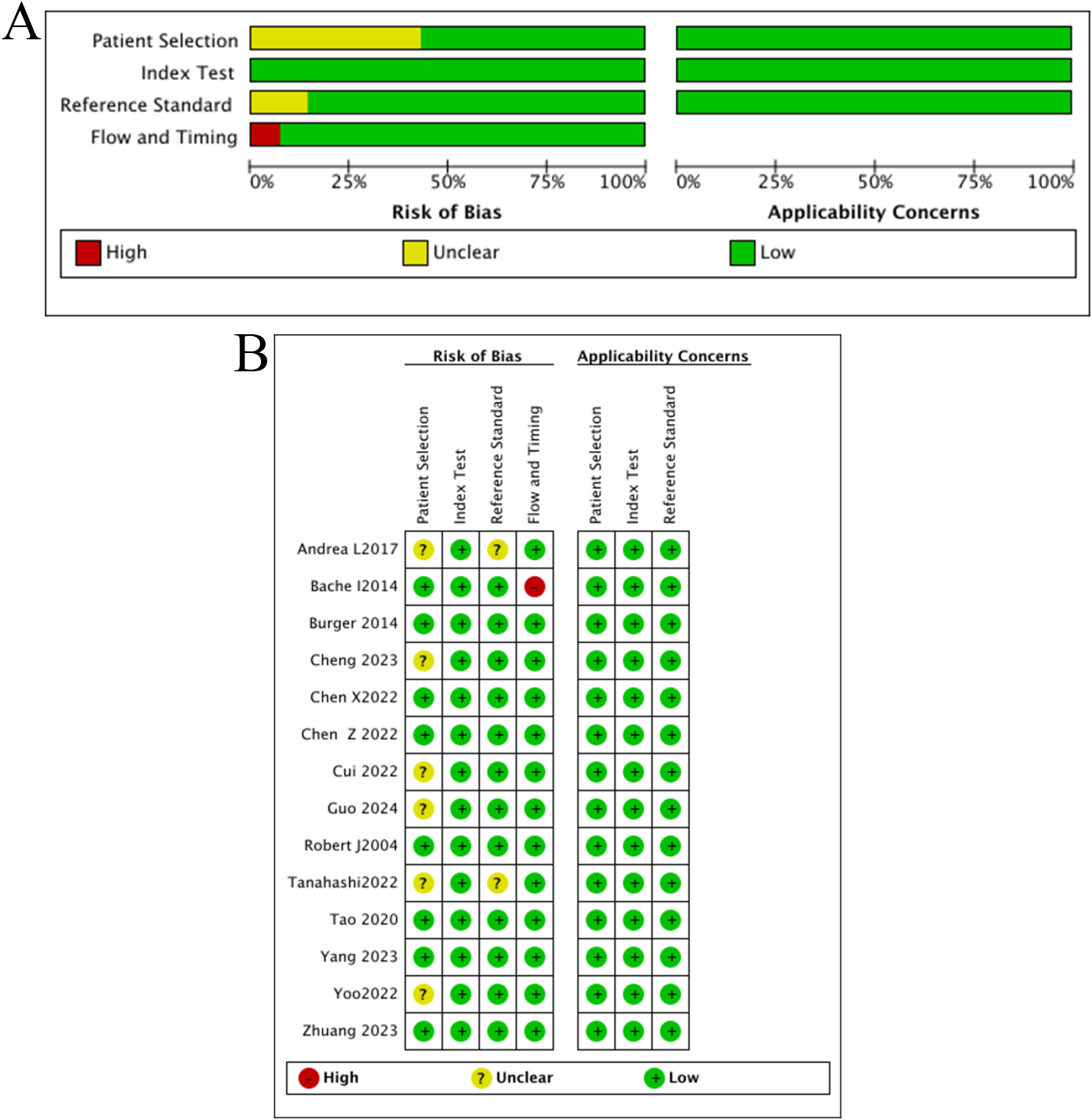
Quality evaluation results **(A)** risk-of-bias and applicability concerns graph; **(B)** risk-of-bias applicability concerns summary.

The quality assessment indicated that all eligible studies possessed relatively high quality overall. However, some studies received classifications of ‘Unclear’ or ‘High’ ROB in specific domains, primarily due to the following reasons: (i) Studies by Andreal, Cui, Cheng et al. (24–29) did not explicitly state whether the enrolled cases were consecutive or random; (ii) The definition of MPR in the studies by Andreal and Tanahashi et al. (28, 29) deviated from the consensus criterion (≤10% viable tumor cells). Andreal et al.’s study defined MPR as ≤1% viable tumor cells, while Tanahashi et al.’s study used a threshold of ≤1/3. Consequently, whether these criteria accurately differentiated the target condition remained uncertain; (iii) In the study by Bahce et al. (30), eight enrolled patients were excluded from the final analysis for various reasons. These issues introduce potential biases that cannot be fully disregarded. The results are presented in **Figure 2**.

### 3.3 Meta-analysis results

#### 3.3.1 Threshold effect and heterogeneity

Spearman correlation analysis revealed correlation coefficients of 0.357 (P = 0.432>0.05) for ΔSUVmax and 0.331 (P = 0.518>0.05) for SUVmax between sensitivity and (1-specificity), suggesting the absence of a threshold effect. Heterogeneity analysis, however, indicated substantial heterogeneity for most pooled estimates. For ΔSUVmax, the I² values were 69.57% for sensitivity, 59.91% for specificity, 36.69% for PLR, 75.68% for NLR, and 99.92% for DOR. For SUVmax, I² values were 83.42% for sensitivity, 4.4% for specificity, 0% for PLR, 88.09% for NLR, and 99.82% for DOR. Given the significant heterogeneity observed for sensitivity, NLR, and DOR in both analyses, a random-effects model was employed for the meta-analysis (**Table 1**).

**Table 1:** 18F-FDG PET/CT prediction of MPR after neoadjuvant therapy in NSCLC.

Pooled effect size			Heterogeneity test		
Pooled effects	Effect value	95%CI	I^2^ (%)	Cochrane Q	*P*
Meta-analysis results (△SUVmax)					
Sensitivity	0.87	0.73-0.94	69.57	19.71	<0.05
Specificity	0.93	0.84-0.97	59.91	14.97	<0.05
PLR	12.81	5.09-32.23	36.69	17.16	<0.05
NLR	0.14	0.07-0.30	75.68	24.67	<0.05
DOR	89.12	22.86-350.44	99.92	7106.20	<0.05
sAUC	0.96	0.94-0.97	–	–	–
Meta-analysis results (SUVmax)					
Sensitivity	0.80	0.63-0.91	83.42	30.16	<0.05
Specificity	0.94	0.87-0.97	4.4	5.23	0.39
PLR	12.98	6.28-26.82	0	5.23	0.38
NLR	0.21	0.1-0.43	88.09	42	<0.05
DOR	62.24	20.78-350.44	99.82	2460	<0.05
sAUC	0.95	0.93-0.96	–	–	–

#### 3.3.2 Pooled effect sizes

**Figure 3:**
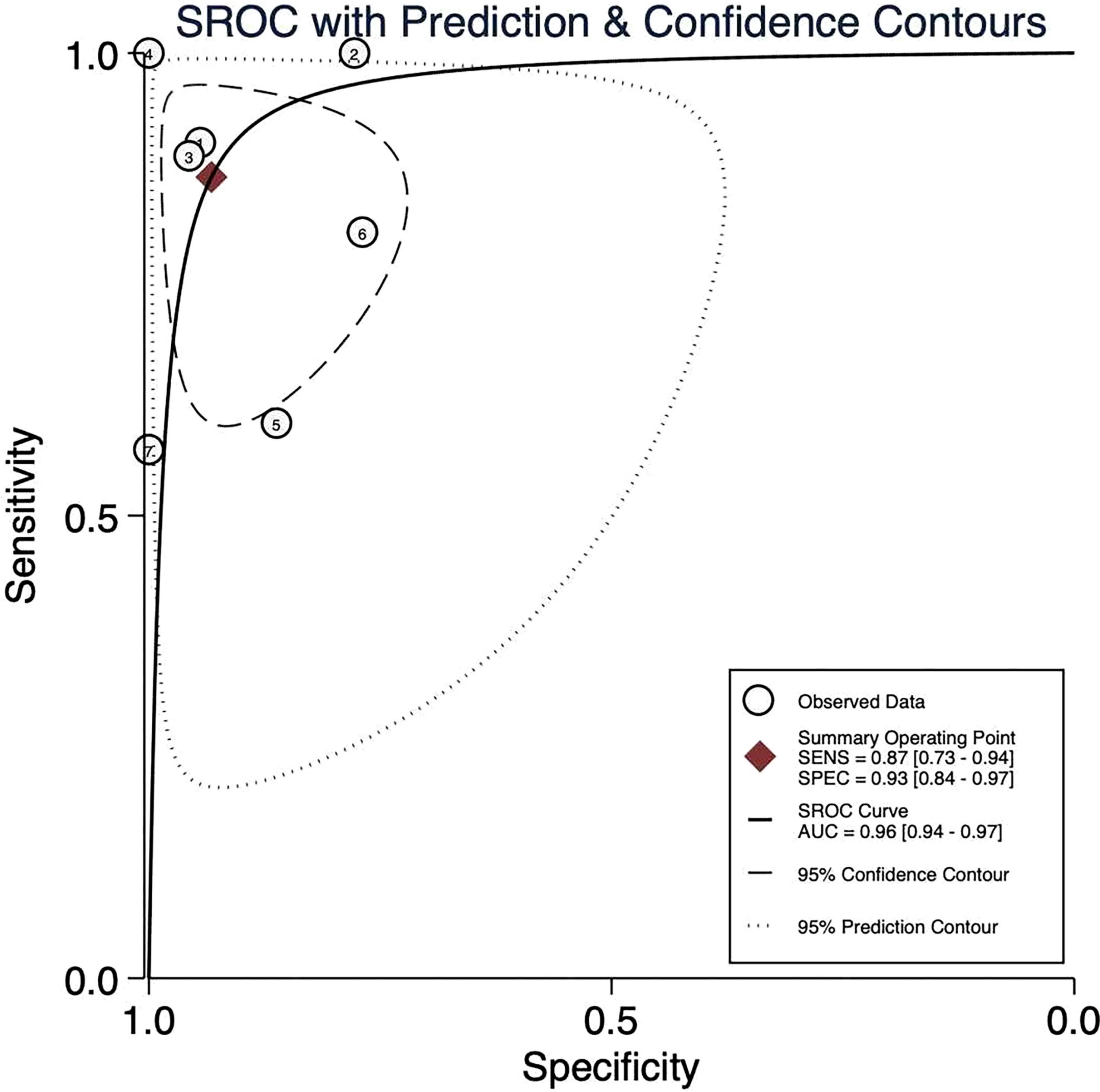
Summary receiver operating characteristic (SROC) curve for ΔSUVmax% in predicting major pathological response (MPR) after neoadjuvant therapy in NSCLC. For ease of identification, the correspondence between each number and the respective study/dataset is provided in **Table 2**.

**Figure 4:**
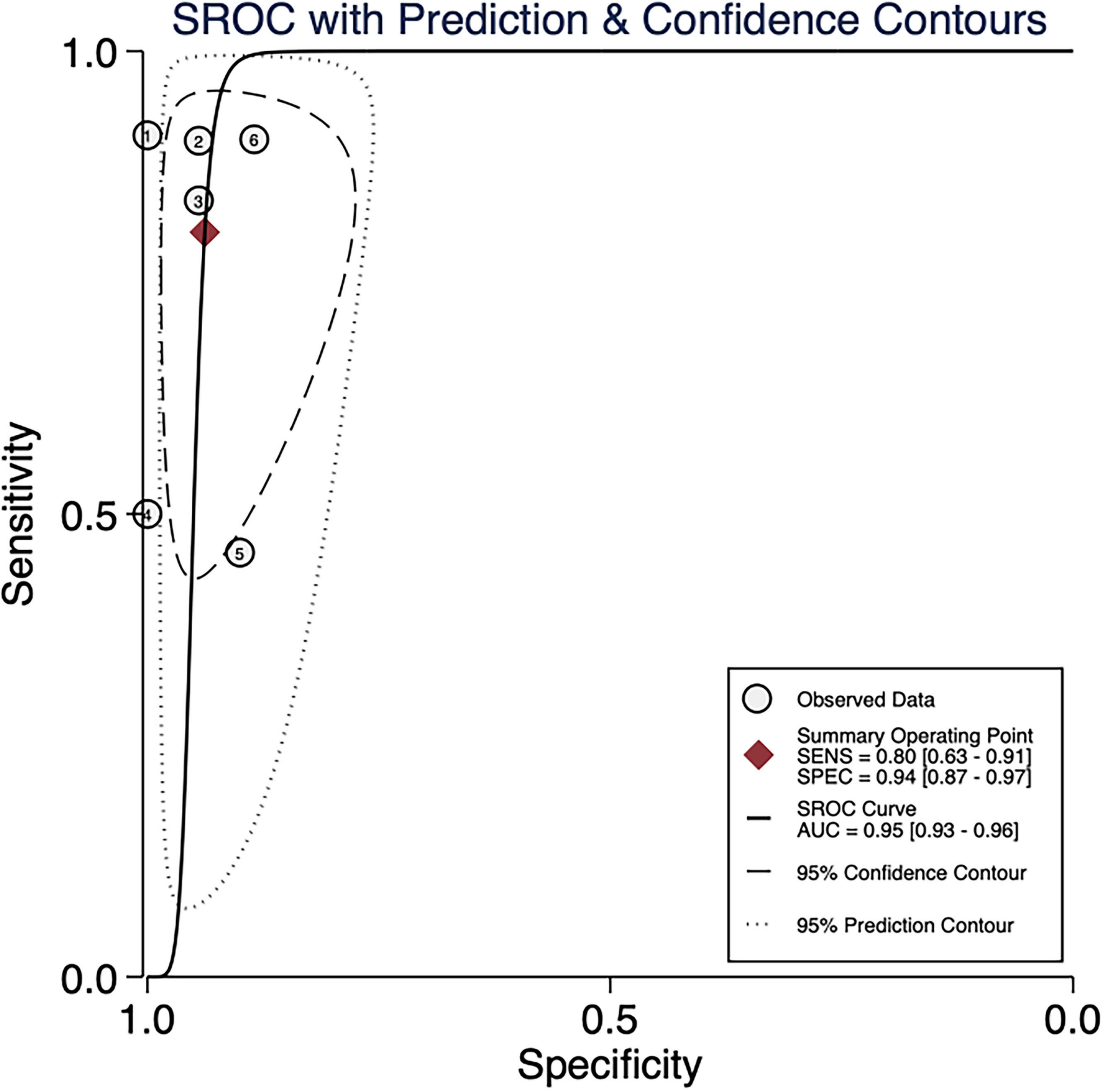
Summary receiver operating characteristic (SROC) curve for SUVmax in predicting major pathological response (MPR) after neoadjuvant therapy in NSCLC. For ease of identification, the correspondence between each number and the respective study/dataset is provided in **Table 2**.

Our meta-analysis yielded the following pooled estimates for predicting MPR using 18F-FDG PET/CT. ΔSUVmax achieved an area under the SROC curve (sAUC) of 0.96 (95% CI: 0.94–0.97), with sensitivity, specificity, PLR, NLR, and DOR of 0.87 (0.73–0.94), 0.93 (0.84–0.97), 12.81 (5.09–32.23), 0.14 (0.07–0.30), and 89.12 (22.86–350.44), respectively. SUVmax exhibited an sAUC of 0.95 (0.93–0.96), and corresponding values of 0.80 (0.63–0.91) for sensitivity, 0.94 (0.87–0.97) for specificity, and 62.24 (22.86–350.44) for DOR. These results demonstrated high diagnostic accuracy for both ΔSUVmax and SUVmax (**Figures 3**, **4**). **Figure 3** includes 7 data points derived from 5 articles, whereas **Figure 4** includes 6 data points derived from 4 articles. Each numbered circle in the figures represents one independently included dataset. When a single study reported multiple independently extractable analytical units, such as different cohorts or different subgroups, these were included as separate data points. Therefore, the number of data points shown in a figure may exceed the number of included articles, and the number of data points may also differ across figures. For ease of identification, the correspondence between each number and the respective study/dataset is provided in **Table 2**.

**Table 2:** Correspondence between numbered points in **Figures 3**, **4**, **6** and **7** and the included studies/datasets.

Point label	Figure(s)	First author, year	Parameter	Note
1	**Figure 3**, **6**	Zhuang 2023 (33)	ΔSUVmax%	cohort A
2	**Figure 3**, **6**	Bahce 2014 (30)	ΔSUVmax%	/
3	**Figure 3**, **6**	Chen 2023 (23)	ΔSUVmax%	(I-C) cohort
4	**Figure 3**, **6**	Chen 2023 (23)	ΔSUVmax%	(I-M) cohort
5	**Figure 3**, **6**	Burger 2015 (31)	ΔSUVmax%	/
6	**Figure 3**, **6**	Chen 2022 (22)	ΔSUVmax%	/
7	**Figure 3**, **6**	Zhuang 2023 (33)	ΔSUVmax%	cohort B
1	**Figure 4**, **7**	Cui 2022 (27)	SUVmax	/
2	**Figure 4**, **7**	Zhuang 2023 (33)	SUVmax	cohort A
3	**Figure 4**, **7**	Zhuang 2023 (33)	SUVmax	cohort C
4	**Figure 4**, **7**	Zhuang 2023 (33)	SUVmax	cohort B
5	**Figure 4**, **7**	Burger 2015 (31)	SUVmax	/
6	**Figure 4**, **7**	Chen 2023 (23)	SUVmax	/

(I-M), ICI monotherapy; (I-C), ICI combination therapy.

#### 3.3.3 Meta-regression and subgroup analyses

**Figure 5:**
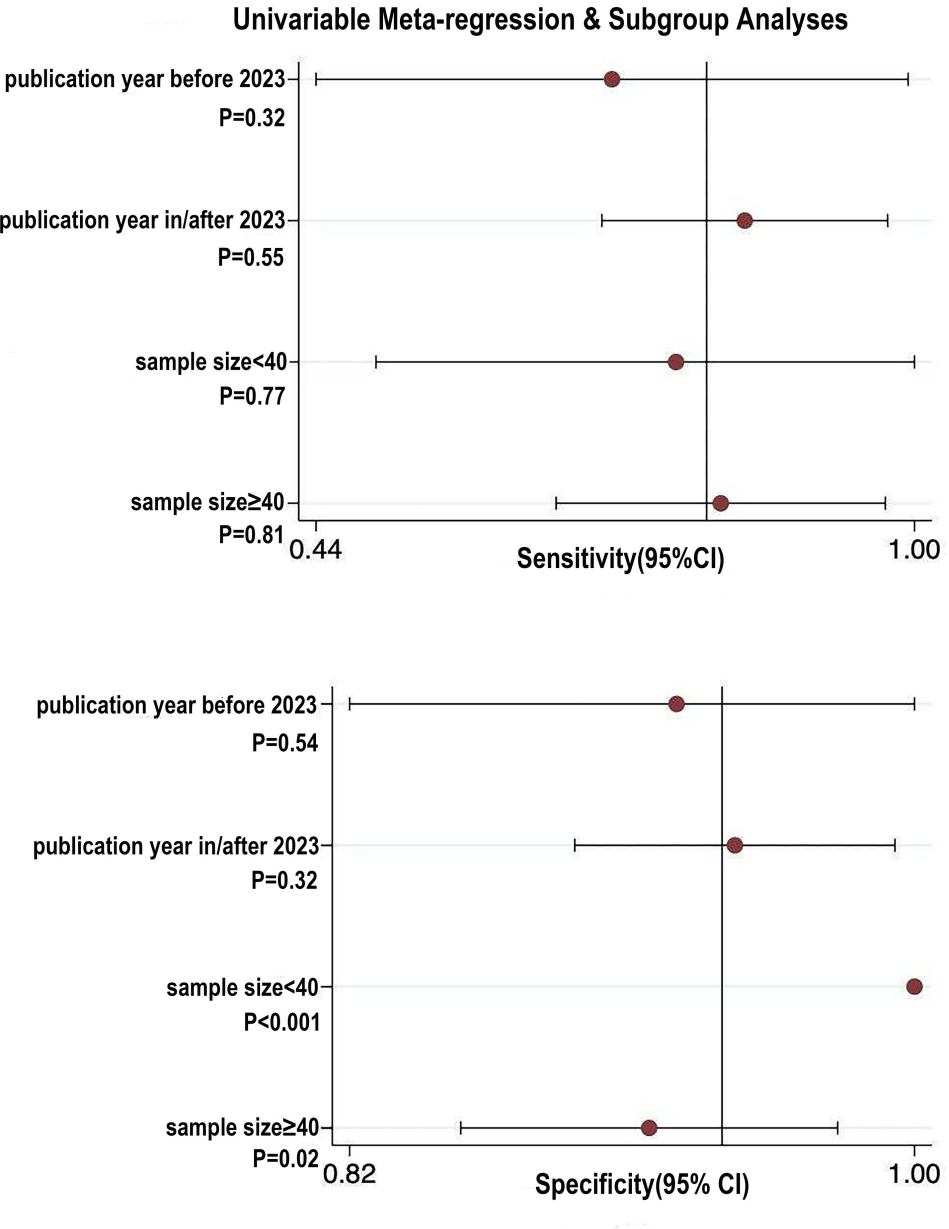
Subgroup/meta-regression plot showing pooled sensitivity and specificity (with 95% confidence intervals) for SUVmax, stratified by publication year and sample size categories.

The results of the I² statistic revealed substantial heterogeneity in the eligible studies. To examine the possible origins, univariate meta-regression and subgroup analyses were implemented utilizing the following covariates: publication year, treatment regimen, sample size, proportion of adenocarcinoma cases, and the cut-off value for ΔSUVmax%. The meta-regression identified that treatment regimen and proportion of adenocarcinoma were significant sources of heterogeneity for pooled sensitivity (P < 0.05), while publication year and ΔSUVmax% cut-off value significantly contributed to heterogeneity in pooled specificity (P < 0.05). These findings suggested that the publication year, treatment regimen, proportion of adenocarcinoma cases, and cutoff value may be potential sources of heterogeneity. Subgroup analyses further elucidated these findings. Studies published after 2020 exhibited higher pooled sensitivity and specificity relative to those before 2020. Studies employing a ΔSUVmax% cut-off >55% showed lower sensitivity and specificity than those using a cut-off ≤55%. The specificity of studies evaluating single-agent neoadjuvant therapy was comparable to that of combination therapy, but the sensitivity was lower. Studies with a proportion of adenocarcinoma ≥40% had lower pooled sensitivity and specificity than those with <40%. Studies with a sample size ≥40 demonstrated lower pooled sensitivity and specificity than smaller studies (<40 subjects) (**Table 3**). For SUVmax, subgroup analyses stratified by sample size and publication year did not reveal statistically significant associations (P ≥ 0.05), leaving the sources of heterogeneity for this parameter unclear (**Figure 5**).

**Table 3:** Subgroup analysis results for △SUVmax%.

Group	Studies	Sensitivity	P	Specificity	P
Publication year					
before 2020	2	0.84	0.73	0.84	0.01
after 2020	5	0.87	0.84	0.95	0.61
Treatment regimen					
Single therapy	4	0.78	0.01	0.94	0.88
Combination therapy	3	0.92	0.76	0.93	0.82
Cut-off					
cut-off<55%	4	0.88	0.96	0.98	0.05
cut-off≧55%	3	0.82	–	0.81	–
Sample size					
<40	4	0.83	0.30	0.89	0.06
≧40	3	0.92	0.45	0.97	0.45
Proportion of adenocarcinoma					
<40%	3	0.76	0.04	0.92	0.53
≧40%	4	0.90	0.68	0.94	0.81

Proportion of adenocarcinoma: The ratio of adenocarcinoma patients to the total sample size; Single therapy: A neoadjuvant treatment regimen comprising only one therapeutic modality; Combination therapy: A treatment regimen incorporating two or more therapeutic modalities.

#### 3.3.4 Publication bias

**Figure 6:**
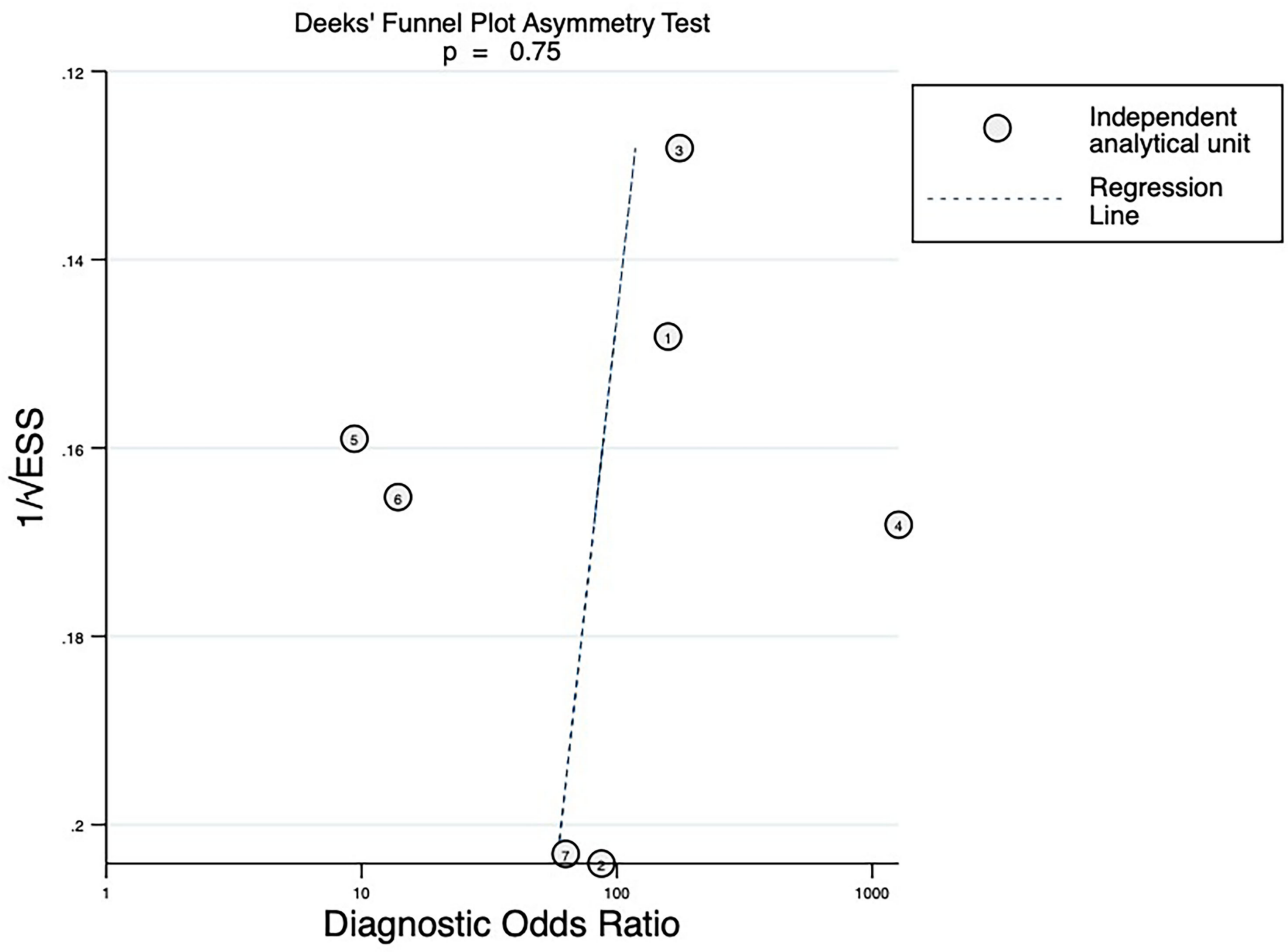
Deeks’ funnel plot asymmetry test assessing publication bias for ΔSUVmax% (each circle represents one independent analytical unit; ESS denotes effective sample size). A total of 7 independent analytical units were derived from 5 articles. For ease of identification, the correspondence between each number and the respective study/dataset is provided in **Table 2**.

**Figure 7:**
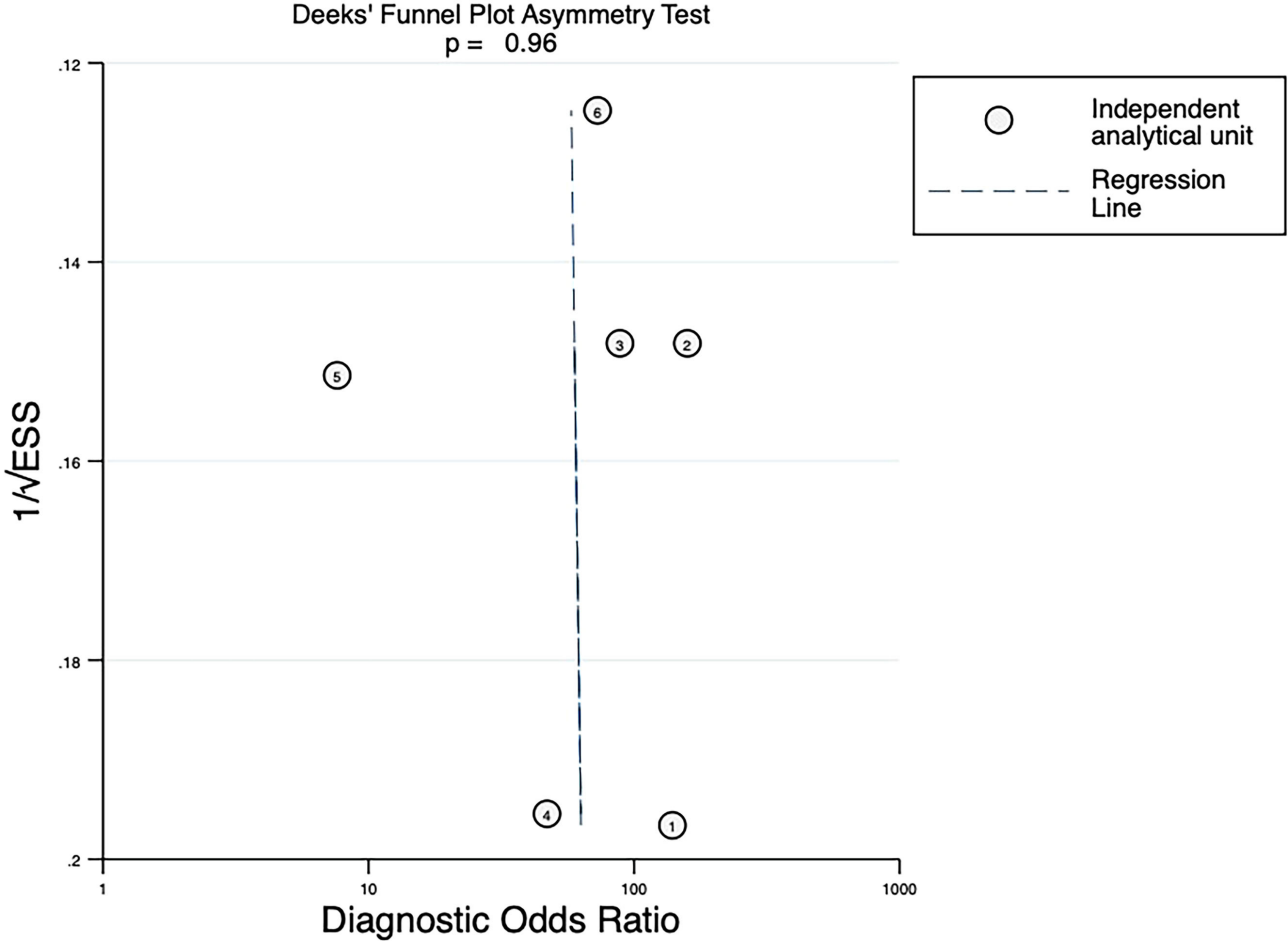
Deeks’ funnel plot asymmetry test assessing publication bias for SUVmax (each circle represents one independent analytical unit; ESS denotes effective sample size). A total of 6 independent analytical units were derived from 4 articles. For ease of identification, the correspondence between each number and the respective study/dataset is provided in **Table 2**.

The Deeks’ funnels revealed no significant publication bias for studies evaluating 18F-FDG PET/CT in predicting pathological response after neoadjuvant therapy in NSCLC, with slope coefficient *P*-values of 0.75 for ΔSUVmax and 0.96 for SUVmax (*P* > 0.05) (**Figure 6**, **7**).

#### 3.3.5 Other PET/CT parameters

Beyond SUVmax and ΔSUVmax%, this systematic review identified several other PET/CT parameters evaluated for predicting pathological response in NSCLC post-neoadjuvant therapy, including total lesion glycolysis (TLG), metabolic tumor volume (MTV), and SULpeak. However, the number of eligible studies for each of these parameters was insufficient (all ≤ 3) to conduct a meaningful quantitative meta-analysis with adequate statistical power. Therefore, a qualitative summary of their findings was provided.

Three studies (21, 24, 27) reported the diagnostic performance of SULpeak. Cui et al. (27) reported a sensitivity, specificity, and AUC of 100%, 80%, and 0.933, respectively, at a cut-off of 2.64. Guo et al. (24), using a similar cut-off of 2.8, found a lower sensitivity (76.9%) but higher specificity (92%) and an AUC of 0.92. Conversely, Tao et al. (21) employed a higher cut-off of 6.7, resulting in a sensitivity of 92.3%, specificity of 86.1%, and AUC of 0.9. Collectively, these studies suggest the potential value of SULpeak.

The studies by Guo and Tao et al. (21, 24) also evaluated MTV, TLG, and their changes (ΔMTV, ΔTLG). Nevertheless, the chosen thresholds for these parameters differed substantially between studies, and their reported performance metrics showed considerable differences, as summarized in **Table 4**.

**Table 4:** Thresholds and diagnostic performance of additional 18F-FDG PET/CT parameters (MTV, TLG, ΔMTV, ΔTLG, and SULpeak) for predicting major pathological response after neoadjuvant therapy in NSCLC.

Study	Threshold	AUC	Sensitivity	Specificity
MTV:				
Tao (21)	16.4	0.74	69.2%	69.6%
Guo (24)	1.9	0.67	84.6%	52%
TLG:				
Tao (21)	87.1	0.84	76.9%	73.9%
Guo (24)	3	0.80	92.3%	52%
△MTV				
Tao (21)	-33%	0.92	92.3%	82.6%
Guo (24)	-63.6%	0.73	57.7%	88%
△TLG				
Tao (21)	-60%	0.99	100%	95.7%
Guo (24)	-90.9%	0.84	69.2%	92%
SULpeak				
Cui (27) Guo (24) Tao (21)	2.64 2.8 6.7	0.93 0.92 0.9	100% 76.9% 92.3%	80% 92% 86.1%

Because no more than three studies were available for each parameter, a quantitative meta-analysis was not performed and the results are summarized qualitatively.

Overall, these preliminary investigations hinted at the potential utility of parameters like SULpeak, MTV, and TLG as predictive biomarkers.

## 4 Discussion

This systematic review and meta-analysis indicated that metabolic parameters derived from 18F-FDG PET/CT, particularly ΔSUVmax% and SUVmax, exhibited high diagnostic accuracy in identifying an MPR after neoadjuvant therapy for resectable NSCLC. The pooled sensitivity and specificity were approximately 0.8–0.9, and the sAUC was 0.95–0.96. Additionally, meta-regression suggested that variation in treatment regimens, histologic composition, and the ΔSUVmax% cutoff partly explained between-study heterogeneity. Exploratory data on SULpeak, MTV, and TLG indicate that these may serve as additional imaging biomarkers for response assessment. However, the evidence is inconclusive.

The high pooled diagnostic performance of ΔSUVmax% indicates that early changes in tumor glucose metabolism reveal biologically significant treatment effects beyond static baseline uptake. By integrating pretreatment metabolic burden with post-treatment residual activity, ΔSUVmax% may reflect the balance between tumor cell eradication and persistence. Across the included studies, a reduction in ΔSUVmax% of approximately 50%–60% (the most commonly used cutoff) was significantly associated with MPR. A moderate difference in pooled sensitivity was observed between ΔSUVmax% and SUVmax. However, their similar sAUC values suggest that baseline metabolic intensity and dynamic metabolic changes are both informative. Compared with SUVmax, ΔSUVmax% showed slightly higher sensitivity with comparable specificity. This suggests that a response-adaptive metric could reduce false-negative classifications among responders while retaining the ability to rule out non-responders. In the neoadjuvant setting, underestimating the response can result in premature treatment escalation or unnecessary delays in surgery; therefore, higher sensitivity is clinically desirable. Conversely, the high positive likelihood ratios of both parameters imply that a strong metabolic response (i.e., low post-treatment SUVmax or a marked decrease in ΔSUVmax%) corresponds to a high probability of MPR.

Meta-regression and subgroup analyses revealed the mechanisms underlying the sources of heterogeneity. Studies using combination regimens (chemoimmunotherapy or chemoradiotherapy) exhibited greater sensitivity to changes in ΔSUVmax% than studies using single-modality neoadjuvant therapy (chemotherapy alone or immunotherapy alone). This suggests that successful multimodal regimens induce more profound metabolic suppression (37, 38). The lower sensitivity observed in single-modality cohorts may reflect partial cytoreduction or differential immune-mediated effects, wherein treatment-induced inflammation complicates the interpretation of post-treatment uptake. Similarly, studies using a ΔSUVmax% cutoff < 55% achieved higher sensitivity and specificity than studies using a higher cutoff. This implies that an overly stringent cutoff may reclassify true responders as non-responders without improving the exclusion of non-responders. Studies with a higher proportion of adenocarcinoma patients demonstrated lower sensitivity and specificity. This is consistent with adenocarcinoma’s typically lower baseline FDG uptake and more heterogeneous metabolic response. These features are often more pronounced in squamous cell carcinoma (39–41). Together, these findings highlight that the performance of 18F-FDG PET/CT depends on the underlying histology and the context of the treatment rather than being uniform across all NSCLC populations. Finally, the relationship between publication years and the evolution of treatment regimens and the standardization of PET/CT techniques and pathological assessment was analyzed. Most studies published after 2020 combined perioperative immunotherapy or chemoimmunotherapy, IASLC-consistent pathological MPR criteria (42), and modern PET/CT protocols (43). These studies reported higher pooled sensitivity and specificity than earlier trials based on chemotherapy or chemoradiotherapy. This effect of publication year likely reflects parallel improvements in systemic regimens, scanner technology, acquisition and reconstruction standardization, and pathology assessment rather than a pure time-related bias.

For evaluating the efficacy of neoadjuvant regimens in resectable NSCLC, the only accepted “gold standard” is pathological assessment of surgical specimens (MPR/pathological complete response [pCR]) (44). Imaging serves as a complement to this assessment, and molecular measures such as ctDNA can provide additional information.

However, neoadjuvant therapy-induced inflammation and tissue repair pose challenges to PET/CT interpretation and predictive accuracy. During therapy, the recruitment of macrophages and other inflammatory cells to the treatment site can affect SUVmax and other 18F-FDG PET/CT parameters (31, 45). This nonspecific uptake may lead to the overestimation of viable tumor cells. Reinhardt et al. demonstrated that FDG uptake is nearly indistinguishable between viable tumor cells and macrophages involved in inflammatory reactions (46). When pseudoprogression is suspected, PET/CT findings should be interpreted in conjunction with a comprehensive clinical assessment and a thorough imaging review. Follow-up is also necessary, and a tissue biopsy should be considered when appropriate. Conclusions should not be based on a single imaging report (47, 48).

Accordingly, we summarize treatment-specific considerations and propose the following recommendations for PET/CT response assessment.

The interval between completion of neoadjuvant therapy and 18F-FDG PET/CT imaging should be carefully selected to minimize the confounding effects of early inflammation. For neoadjuvant regimens involving chemotherapy or chemoimmunotherapy, early reductions in SUVmax and ΔSUVmax% after cycles two to four have consistently predicted MPR, suggesting the feasibility of metabolic response assessment in this context. Regimens including thoracic radiotherapy often require an interval of about four weeks or longer. Some studies suggest that, in these settings, PET/CT after radiotherapy may require an even longer delay than in other neoadjuvant settings (32). A pragmatic approach is to schedule a metabolic response assessment two to four weeks after chemotherapy or chemoimmunotherapy and defer imaging until at least four weeks after thoracic radiotherapy. The optimal timing should be confirmed in future prospective studies.

Additionally, radiomics and machine-learning approaches should be applied to 18F-FDG PET/CT, where feasible. Recent small pilot series and multicenter studies suggest that PET-based radiomic features can stratify patients by their likelihood of MPR or pCR after neoadjuvant immunochemotherapy. These features may also outperform conventional SUV metrics for predicting pCR after neoadjuvant chemoradiotherapy (26, 27, 34). These findings complement the present meta-analysis and imply that SUVmax and ΔSUVmax% are first-generation biomarkers that are readily available. However, high-dimensional radiomic models could offer additional value in specialized centers. In addition, systematic reviews of PET/CT in immunotherapy underscore the need to interpret metabolic changes in the context of immune-related phenomena such as pseudoprogression and immune activation.

Furthermore, promising parameters such as SULpeak, MTV, and TLG should be explored (49–51). However, the number of available studies remains limited. Additional prospective data are needed to derive robust pooled estimates.

The high sAUC values, PLR > 10, and low NLR observed in this study suggest that 18F-FDG PET/CT has excellent rule-in capability and good rule-out performance for MPR. These results support the integration of these parameters as noninvasive biomarkers into neoadjuvant NSCLC workflows. Pre-treatment and early post-treatment PET/CT can stratify patients into high- and low-probability MPR groups (47). Such stratification can inform multidisciplinary decisions regarding the duration and intensity of neoadjuvant therapy, the timing of surgery, and whether to escalate or de-escalate treatment strategies (52). For example, patients with a minimal metabolic response could be considered for intensified systemic therapy or enrollment in adaptive trials. In contrast, those with a significant metabolic response and an acceptable surgical risk could proceed directly to resection, thereby avoiding unnecessary treatment cycles.

Second, a PET/CT-based response assessment could make clinical trials more efficient by identifying populations likely to achieve MPR or derive long-term benefits. In perioperative immunotherapy trials, incorporating ΔSUVmax% or SUVmax as intermediate endpoints could reduce follow-up requirements and allow for adaptive trial designs. However, the relationships among metabolic response, MPR, and survival must first be prospectively validated (53, 54). Compared with purely anatomical criteria, such as RECIST, metabolic metrics can capture early functional changes and are less affected by fibrosis or treatment-related edema. These features are particularly important in the immunotherapy era (55, 56).

Third, our results suggest that PET/CT can complement emerging biomarkers, such as PD-L1 expression, tumor mutational burden, ctDNA clearance, and immune gene signatures (57, 58). While tissue and blood biomarkers can provide insight into tumor biology and systemic immune status, PET/CT provides whole-body spatial mapping of metabolic response, including occult sites of disease that are inaccessible for pathological sampling (59). Ultimately, integrated models combining metabolic, molecular, and radiomic features may yield more accurate and robust predictions of benefit from neoadjuvant therapy than any single modality alone (60).

Several limitations should be acknowledged. Although only 14 diagnostic studies met our eligibility criteria, these studies included 1,315 patients and represent the most comprehensive dataset currently available for predicting post-neoadjuvant MPR in NSCLC using PET/CT. The sample size was sufficient for random-effects pooling and exploratory meta-regression. However, the findings should be regarded as hypothesis-generating and require confirmation in larger prospective multicenter cohorts. Existing data were insufficient to generate separate pooled estimates for regimens such as chemotherapy alone, chemoimmunotherapy alone, or targeted therapy alone. Consequently, subgroup analyses could only compare single-modality versus combination regimens. All combinations (chemoimmunotherapy, chemoradiotherapy, and immunotherapy plus targeted therapy) were grouped as “combination therapy”. Future meta-analyses and prospective studies should stratify by more detailed treatment categories. Additionally, the inclusion of only English-language publications raised the possibility of language and publication bias. An ROB assessment revealed that some studies had a high risk of selection bias, which could affect the credibility and robustness of the pooled results. Finally, our analysis focused on diagnostic accuracy for pathological response, and survival outcomes were insufficiently reported. Therefore, a formal meta-analysis could not be performed for the associations among PET-derived metrics, MPR, and long-term outcomes.

## 5 Conclusion

In conclusion, this meta-analysis indicates that ΔSUVmax% and SUVmax derived from 18F-FDG PET/CT demonstrate high diagnostic performance in identifying MPR after neoadjuvant therapy in NSCLC. Additionally, this analysis identifies study-level factors that contribute to performance heterogeneity. When interpreted within standardized acquisition and pathological assessment frameworks, these metabolic parameters may serve as noninvasive, complementary tools for assessing responses to support individualized neoadjuvant strategies and enrich clinical trials in the era of perioperative immunotherapy. However, given the limited number of available studies and their predominantly retrospective designs, these findings should be considered hypothesis-generating and require confirmation in large, prospective, multicenter trials.

## Data Availability

The original contributions presented in the study are included in the article/**Supplementary Material**. Further inquiries can be directed to the corresponding authors.

## Supplementary material

Supplementary Table 1 Search Strategy.

Supplementary Table 2 Demographic and baseline clinical features of the subjects.
