# Correction: 18F-FDG PET/CT for predicting major pathological response to neoadjuvant therapy in non-small cell lung cancer: a meta-analysis

**DOI:** 10.3389/fonc.2026.1927780

**Published:** 2026-08-03

**Authors:** Yaoyu Wu, Boxiao Hu, Dazhi Liu, Hao Meng

**Affiliations:** Department of Thoracic Surgery, Northern Theater General Hospital, Shenyang, Liaoning, China

**Keywords:** 18F-FDG, MPR, neoadjuvant therapy, NSCLC, PET/CT, SUVmax

In the published article, there was an omission in the **Funding** statement. The funder “Application Study of AI-Omics and 3D Imaging-Assisted RATS and TNT in the Treatment of Pulmonary Nodules (Grant No. ZZKY2024059)” was erroneously omitted. The correct Funding statement appears below:

*“*The author(s) declared that financial support was received for this work and/or its publication. This work was supported by the Liaoning Province Natural Science Foundation Fund Project (Grant No. 2025-MSLH-718) and the Application Study of AI-Omics and 3D Imaging-Assisted RATS and TNT in the Treatment of Pulmonary Nodules (Grant No. ZZKY2024059).”

The original version of this article has been updated.

