# Supplementary material for: 18F-FDG PET/CT for predicting major pathological response to neoadjuvant therapy in non-small cell lung cancer: a meta-analysis
Source: Front Oncol. 2026 May 15;16:1730878. doi: 10.3389/fonc.2026.1730878 (PMC13218960; doi:10.3389/fonc.2026.1730878)
Supplement: Supplementary Table 1 — Search Strategy. [file Table1.docx]

**Supplementary Table 1:** Seach Strategy.

**PubMed**

| Search number | Query | Sort By | Filters | Search Details | Results |
| --- | --- | --- | --- | --- | --- |
| 1 | "Neoadjuvant Therapy"[Mesh] | Most Recent |  | "Neoadjuvant Therapy"[MeSH Terms] | 31,302 |
| 2 | "neo adjuvant therapy" OR "neo adjuvant treatment" OR "Neoadjuvant Chemoradiation" OR "Neoadjuvant Chemoradiation Therap*" OR "Neoadjuvant Chemoradiation Treatment*" OR "Neoadjuvant Chemoradiations" OR "Neoadjuvant Chemoradiotherapies" OR "Neoadjuvant Chemoradiotherapy" OR "Neoadjuvant Chemotherapies" OR "Neoadjuvant Chemotherapy" OR "Neoadjuvant Chemotherapy Treatment*" OR "Neoadjuvant Radiation" OR "Neoadjuvant Radiation Therap*" OR "Neoadjuvant Radiation Treatment*" OR "Neoadjuvant Radiations" OR "Neoadjuvant Radiotherap*" OR "Neoadjuvant Systemic Therapies" OR "Neoadjuvant Systemic Therapy" OR "Neoadjuvant Systemic Treatment*" OR "Neoadjuvant Therapies" OR "neoadjuvant therapy" OR "neoadjuvant treatment" OR "Neoadjuvant Treatments" |  |  | "neo adjuvant therapy"[All Fields] OR "neo adjuvant treatment"[All Fields] OR "Neoadjuvant Chemoradiation"[All Fields] OR "neoadjuvant chemoradiation therap*"[All Fields] OR "neoadjuvant chemoradiation treatment*"[All Fields] OR ("neoadjuvant therapy"[MeSH Terms] OR ("neoadjuvant"[All Fields] AND "therapy"[All Fields]) OR "neoadjuvant therapy"[All Fields] OR ("neoadjuvant"[All Fields] AND "chemoradiations"[All Fields])) OR "Neoadjuvant Chemoradiotherapies"[All Fields] OR "Neoadjuvant Chemoradiotherapy"[All Fields] OR "Neoadjuvant Chemotherapies"[All Fields] OR "Neoadjuvant Chemotherapy"[All Fields] OR "neoadjuvant chemotherapy treatment*"[All Fields] OR "Neoadjuvant Radiation"[All Fields] OR "neoadjuvant radiation therap*"[All Fields] OR "neoadjuvant radiation treatment*"[All Fields] OR ("neoadjuvant therapy"[MeSH Terms] OR ("neoadjuvant"[All Fields] AND "therapy"[All Fields]) OR "neoadjuvant therapy"[All Fields] OR ("neoadjuvant"[All Fields] AND "radiations"[All Fields])) OR "neoadjuvant radiotherap*"[All Fields] OR "Neoadjuvant Systemic Therapies"[All Fields] OR "Neoadjuvant Systemic Therapy"[All Fields] OR "neoadjuvant systemic treatment*"[All Fields] OR "Neoadjuvant Therapies"[All Fields] OR "neoadjuvant therapy"[All Fields] OR "neoadjuvant treatment"[All Fields] OR "Neoadjuvant Treatments"[All Fields] | 59,963 |
| 3 | "Carcinoma, Non-Small-Cell Lung"[Mesh] | Most Recent |  | "carcinoma, non small cell lung"[MeSH Terms] | 76,131 |
| 4 | "bronchial non small cell cancer"[Title/Abstract] OR "bronchial non small cell carcinoma"[Title/Abstract] OR "lung non small cell cancer"[Title/Abstract] OR "lung non small cell carcinoma"[Title/Abstract] OR "non oat cell lung cancer"[Title/Abstract] OR "non small cell bronchial cancer"[Title/Abstract] OR "Non Small Cell Lung Cancer"[Title/Abstract] OR "non small cell lung carcinoma*"[Title/Abstract] OR "non small cell pulmonary cancer"[Title/Abstract] OR "non small cell pulmonary carcinoma"[Title/Abstract] OR "non squamous NSCLC"[Title/Abstract] OR "nonsmall cell carcinoma of the lung"[Title/Abstract] OR "nonsmall cell lung cancer"[Title/Abstract] OR "nonsmall cell lung carcinoma"[Title/Abstract] OR "pulmonary non small cell cancer"[Title/Abstract] OR "pulmonary non small cell carcinoma"[Title/Abstract] |  |  | "lung non small cell cancer"[Title/Abstract] OR "lung non small cell carcinoma"[Title/Abstract] OR "non oat cell lung cancer"[Title/Abstract] OR "non small cell bronchial cancer"[Title/Abstract] OR "Non Small Cell Lung Cancer"[Title/Abstract] OR "non small cell lung carcinoma*"[Title/Abstract] OR "non small cell pulmonary cancer"[Title/Abstract] OR "non small cell pulmonary carcinoma"[Title/Abstract] OR "non squamous NSCLC"[Title/Abstract] OR "nonsmall cell lung cancer"[Title/Abstract] OR "nonsmall cell lung carcinoma"[Title/Abstract] OR "pulmonary non small cell carcinoma"[Title/Abstract] | 92,557 |
| 5 | (("Fluorodeoxyglucose F18"[Mesh] OR "fluorodeoxyglucose F18-maleimidehexyloxime" [Supplementary Concept]) OR ( "Positron-Emission Tomography"[Mesh] OR "Positron Emission Tomography Computed Tomography"[Mesh] )) OR ( "Single Photon Emission Computed Tomography Computed Tomography"[Mesh] OR "Tomography, X-Ray Computed"[Mesh] ) | Most Recent |  | "Fluorodeoxyglucose F18"[MeSH Terms] OR "fluorodeoxyglucose F18-maleimidehexyloxime"[Supplementary Concept] OR "Positron-Emission Tomography"[MeSH Terms] OR "Positron Emission Tomography Computed Tomography"[MeSH Terms] OR "Single Photon Emission Computed Tomography Computed Tomography"[MeSH Terms] OR "tomography, x ray computed"[MeSH Terms] | 561,000 |
| 6 | "18F FDG"[Title/Abstract] OR "18F Fluorodeoxyglucose"[Title/Abstract] OR "18FDG"[Title/Abstract] OR "2 deoxy 2 fluoro d glucose f 18"[Title/Abstract] OR "2 deoxy 2 fluoro dextro glucose f 18"[Title/Abstract] OR "2 deoxy 2 fluoro glucose f 18"[Title/Abstract] OR "2 deoxy 2 fluoroglucose f 18"[Title/Abstract] OR "2 deoxy d glucose f 18"[Title/Abstract] OR "2 deoxy dextro glucose f 18"[Title/Abstract] OR "2 deoxyfluoroglucose f 18"[Title/Abstract] OR "2 deoxyglucose f 18"[Title/Abstract] OR "2 Fluoro 2 deoxy D glucose"[Title/Abstract] OR "2 fluoro 2 deoxy d glucose f 18"[Title/Abstract] OR "2 Fluoro 2 deoxyglucose"[Title/Abstract] OR "2 fluoro 2 deoxyglucose f 18"[Title/Abstract] OR "2 fluorodeoxyglucose f 18"[Title/Abstract] OR "CAT scan"[Title/Abstract] OR "CAT scanning"[Title/Abstract] OR "Cine CT"[Title/Abstract] OR "computed axial tomography"[Title/Abstract] OR "computed tomographic scan"[Title/Abstract] OR "Computed Tomography"[Title/Abstract] OR "computed tomography scan"[Title/Abstract] OR "Computed X Ray Tomography"[Title/Abstract] OR "computer assisted tomography"[Title/Abstract] OR "computer tomography"[Title/Abstract] OR "computerised axial tomography"[Title/Abstract] OR "computerised tomography"[Title/Abstract] OR "computerized axial tomography"[Title/Abstract] OR "computerized tomography"[Title/Abstract] OR "computerized tomography scan"[Title/Abstract] OR "CT PET"[Title/Abstract] OR "CT PET Scan*"[Title/Abstract] OR "CT SPECT"[Title/Abstract] OR "CT SPECT Scan*"[Title/Abstract] OR "CT SPECTs"[Title/Abstract] OR "CT X Ray*"[Title/Abstract] OR "deoxyfluoroglucose f 18"[Title/Abstract] OR "deoxyglucose f 18"[Title/Abstract] OR "Electron Beam Computed Tomography"[Title/Abstract] OR "Electron Beam Tomography"[Title/Abstract] OR "fludeoxyglucose f 18"[Title/Abstract] OR "fludeoxyglucose f18"[Title/Abstract] OR "fluor 18 FDG"[Title/Abstract] OR "Fluorine 18 fluorodeoxyglucose"[Title/Abstract] OR "fluoro 2 deoxy d glucose f 18"[Title/Abstract] OR "fluoro 2 deoxyglucose f 18"[Title/Abstract] OR "fluorodeoxy d glucose f 18"[Title/Abstract] OR "Fluorodeoxyglucose F 18"[Title/Abstract] OR "Fluorodeoxyglucose F18"[Title/Abstract] OR "glucotrace"[Title/Abstract] OR "meta trace fdg"[Title/Abstract] OR "p.e.t."[Title/Abstract] OR "PET CT"[Title/Abstract] OR "PET CT Scan*"[Title/Abstract] OR "PET Imaging*"[Title/Abstract] OR "PET Scan*"[Title/Abstract] OR "positron emission tomographic scan"[Title/Abstract] OR "positron emission tomographic scanning"[Title/Abstract] OR "Positron Emission Tomography"[Title/Abstract] OR "Positron Emission Tomography Computed Tomography"[Title/Abstract] OR "Positron Emission Tomography Imaging*"[Title/Abstract] OR "positron tomography"[Title/Abstract] OR "Single Photon Emission Computed Tomography Computed Tomography"[Title/Abstract] OR "SPECT CT"[Title/Abstract] OR "SPECT CT Scan*"[Title/Abstract] OR "steripet"[Title/Abstract] OR "Tomodensitometry"[Title/Abstract] OR "Transmission Computed Tomography"[Title/Abstract] OR "X Ray CAT Scan*"[Title/Abstract] OR "X Ray Computed Tomography"[Title/Abstract] OR "X Ray Computer Assisted Tomography"[Title/Abstract] OR "X Ray Computerized Axial Tomography"[Title/Abstract] OR "X Ray Computerized Tomography"[Title/Abstract] OR "X Ray CT Scan*"[Title/Abstract] OR "Xray Computed Tomography"[Title/Abstract] |  |  | "18F FDG"[Title/Abstract] OR "18F Fluorodeoxyglucose"[Title/Abstract] OR "18FDG"[Title/Abstract] OR "2 Fluoro 2 deoxy D glucose"[Title/Abstract] OR "2 fluoro 2 deoxy d glucose f 18"[Title/Abstract] OR "2 Fluoro 2 deoxyglucose"[Title/Abstract] OR "CAT scan"[Title/Abstract] OR "CAT scanning"[Title/Abstract] OR "Cine CT"[Title/Abstract] OR "computed axial tomography"[Title/Abstract] OR "computed tomographic scan"[Title/Abstract] OR "Computed Tomography"[Title/Abstract] OR "computed tomography scan"[Title/Abstract] OR "Computed X Ray Tomography"[Title/Abstract] OR "computer assisted tomography"[Title/Abstract] OR "computer tomography"[Title/Abstract] OR "computerised axial tomography"[Title/Abstract] OR "computerised tomography"[Title/Abstract] OR "computerized axial tomography"[Title/Abstract] OR "computerized tomography"[Title/Abstract] OR "computerized tomography scan"[Title/Abstract] OR "CT PET"[Title/Abstract] OR "ct pet scan*"[Title/Abstract] OR "CT SPECT"[Title/Abstract] OR "ct spect scan*"[Title/Abstract] OR "ct x ray*"[Title/Abstract] OR "Electron Beam Computed Tomography"[Title/Abstract] OR "Electron Beam Tomography"[Title/Abstract] OR "fludeoxyglucose f 18"[Title/Abstract] OR "fludeoxyglucose f18"[Title/Abstract] OR "fluor 18 FDG"[Title/Abstract] OR "Fluorine 18 fluorodeoxyglucose"[Title/Abstract] OR "Fluorodeoxyglucose F 18"[Title/Abstract] OR "Fluorodeoxyglucose F18"[Title/Abstract] OR "p e t"[Title/Abstract] OR "PET CT"[Title/Abstract] OR "pet ct scan*"[Title/Abstract] OR "pet imaging*"[Title/Abstract] OR "pet scan*"[Title/Abstract] OR "positron emission tomographic scan"[Title/Abstract] OR "positron emission tomographic scanning"[Title/Abstract] OR "Positron Emission Tomography"[Title/Abstract] OR "Positron Emission Tomography Computed Tomography"[Title/Abstract] OR "positron emission tomography imaging*"[Title/Abstract] OR "positron tomography"[Title/Abstract] OR "Single Photon Emission Computed Tomography Computed Tomography"[Title/Abstract] OR "SPECT CT"[Title/Abstract] OR "spect ct scan*"[Title/Abstract] OR "Tomodensitometry"[Title/Abstract] OR "Transmission Computed Tomography"[Title/Abstract] OR "x ray cat scan*"[Title/Abstract] OR "X Ray Computed Tomography"[Title/Abstract] OR "X Ray Computer Assisted Tomography"[Title/Abstract] OR "X Ray Computerized Axial Tomography"[Title/Abstract] OR "X Ray Computerized Tomography"[Title/Abstract] OR "x ray ct scan*"[Title/Abstract] OR "Xray Computed Tomography"[Title/Abstract] | 483,930 |
| 7 | #1 OR #2 |  |  | "Neoadjuvant Therapy"[MeSH Terms] OR ("neo adjuvant therapy"[All Fields] OR "neo adjuvant treatment"[All Fields] OR "Neoadjuvant Chemoradiation"[All Fields] OR "neoadjuvant chemoradiation therap*"[All Fields] OR "neoadjuvant chemoradiation treatment*"[All Fields] OR ("Neoadjuvant Therapy"[MeSH Terms] OR ("neoadjuvant"[All Fields] AND "therapy"[All Fields]) OR "Neoadjuvant Therapy"[All Fields] OR ("neoadjuvant"[All Fields] AND "chemoradiations"[All Fields])) OR "Neoadjuvant Chemoradiotherapies"[All Fields] OR "Neoadjuvant Chemoradiotherapy"[All Fields] OR "Neoadjuvant Chemotherapies"[All Fields] OR "Neoadjuvant Chemotherapy"[All Fields] OR "neoadjuvant chemotherapy treatment*"[All Fields] OR "Neoadjuvant Radiation"[All Fields] OR "neoadjuvant radiation therap*"[All Fields] OR "neoadjuvant radiation treatment*"[All Fields] OR ("Neoadjuvant Therapy"[MeSH Terms] OR ("neoadjuvant"[All Fields] AND "therapy"[All Fields]) OR "Neoadjuvant Therapy"[All Fields] OR ("neoadjuvant"[All Fields] AND "radiations"[All Fields])) OR "neoadjuvant radiotherap*"[All Fields] OR "Neoadjuvant Systemic Therapies"[All Fields] OR "Neoadjuvant Systemic Therapy"[All Fields] OR "neoadjuvant systemic treatment*"[All Fields] OR "Neoadjuvant Therapies"[All Fields] OR "Neoadjuvant Therapy"[All Fields] OR "neoadjuvant treatment"[All Fields] OR "Neoadjuvant Treatments"[All Fields]) | 59,963 |
| 8 | #3 OR #4 |  |  | "carcinoma, non small cell lung"[MeSH Terms] OR "lung non small cell cancer"[Title/Abstract] OR "lung non small cell carcinoma"[Title/Abstract] OR "non oat cell lung cancer"[Title/Abstract] OR "non small cell bronchial cancer"[Title/Abstract] OR "Non Small Cell Lung Cancer"[Title/Abstract] OR "non small cell lung carcinoma*"[Title/Abstract] OR "non small cell pulmonary cancer"[Title/Abstract] OR "non small cell pulmonary carcinoma"[Title/Abstract] OR "non squamous NSCLC"[Title/Abstract] OR "nonsmall cell lung cancer"[Title/Abstract] OR "nonsmall cell lung carcinoma"[Title/Abstract] OR "pulmonary non small cell carcinoma"[Title/Abstract] | 106,943 |
| 9 | #5 OR #6 |  |  | "Fluorodeoxyglucose F18"[MeSH Terms] OR "fluorodeoxyglucose F18-maleimidehexyloxime"[Supplementary Concept] OR "positron emission tomography"[MeSH Terms] OR "Positron Emission Tomography Computed Tomography"[MeSH Terms] OR "Single Photon Emission Computed Tomography Computed Tomography"[MeSH Terms] OR "tomography, x ray computed"[MeSH Terms] OR "18F FDG"[Title/Abstract] OR "18F Fluorodeoxyglucose"[Title/Abstract] OR "18FDG"[Title/Abstract] OR "2 Fluoro 2 deoxy D glucose"[Title/Abstract] OR "2 fluoro 2 deoxy d glucose f 18"[Title/Abstract] OR "2 Fluoro 2 deoxyglucose"[Title/Abstract] OR "CAT scan"[Title/Abstract] OR "CAT scanning"[Title/Abstract] OR "Cine CT"[Title/Abstract] OR "computed axial tomography"[Title/Abstract] OR "computed tomographic scan"[Title/Abstract] OR "Computed Tomography"[Title/Abstract] OR "computed tomography scan"[Title/Abstract] OR "Computed X Ray Tomography"[Title/Abstract] OR "computer assisted tomography"[Title/Abstract] OR "computer tomography"[Title/Abstract] OR "computerised axial tomography"[Title/Abstract] OR "computerised tomography"[Title/Abstract] OR "computerized axial tomography"[Title/Abstract] OR "computerized tomography"[Title/Abstract] OR "computerized tomography scan"[Title/Abstract] OR "CT PET"[Title/Abstract] OR "ct pet scan*"[Title/Abstract] OR "CT SPECT"[Title/Abstract] OR "ct spect scan*"[Title/Abstract] OR "ct x ray*"[Title/Abstract] OR "Electron Beam Computed Tomography"[Title/Abstract] OR "Electron Beam Tomography"[Title/Abstract] OR "fludeoxyglucose f 18"[Title/Abstract] OR "fludeoxyglucose f18"[Title/Abstract] OR "fluor 18 FDG"[Title/Abstract] OR "Fluorine 18 fluorodeoxyglucose"[Title/Abstract] OR "Fluorodeoxyglucose F 18"[Title/Abstract] OR "Fluorodeoxyglucose F18"[Title/Abstract] OR "p e t"[Title/Abstract] OR "PET CT"[Title/Abstract] OR "pet ct scan*"[Title/Abstract] OR "pet imaging*"[Title/Abstract] OR "pet scan*"[Title/Abstract] OR "positron emission tomographic scan"[Title/Abstract] OR "positron emission tomographic scanning"[Title/Abstract] OR "positron emission tomography"[Title/Abstract] OR "Positron Emission Tomography Computed Tomography"[Title/Abstract] OR "positron emission tomography imaging*"[Title/Abstract] OR "positron tomography"[Title/Abstract] OR "Single Photon Emission Computed Tomography Computed Tomography"[Title/Abstract] OR "SPECT CT"[Title/Abstract] OR "spect ct scan*"[Title/Abstract] OR "Tomodensitometry"[Title/Abstract] OR "Transmission Computed Tomography"[Title/Abstract] OR "x ray cat scan*"[Title/Abstract] OR "X Ray Computed Tomography"[Title/Abstract] OR "X Ray Computer Assisted Tomography"[Title/Abstract] OR "X Ray Computerized Axial Tomography"[Title/Abstract] OR "X Ray Computerized Tomography"[Title/Abstract] OR "x ray ct scan*"[Title/Abstract] OR "Xray Computed Tomography"[Title/Abstract] | 793,527 |
| 10 | #7 AND #8 AND #9 |  |  | ("Neoadjuvant Therapy"[MeSH Terms] OR ("neo adjuvant therapy"[All Fields] OR "neo adjuvant treatment"[All Fields] OR "Neoadjuvant Chemoradiation"[All Fields] OR "neoadjuvant chemoradiation therap*"[All Fields] OR "neoadjuvant chemoradiation treatment*"[All Fields] OR ("Neoadjuvant Therapy"[MeSH Terms] OR ("neoadjuvant"[All Fields] AND "therapy"[All Fields]) OR "Neoadjuvant Therapy"[All Fields] OR ("neoadjuvant"[All Fields] AND "chemoradiations"[All Fields])) OR "Neoadjuvant Chemoradiotherapies"[All Fields] OR "Neoadjuvant Chemoradiotherapy"[All Fields] OR "Neoadjuvant Chemotherapies"[All Fields] OR "Neoadjuvant Chemotherapy"[All Fields] OR "neoadjuvant chemotherapy treatment*"[All Fields] OR "Neoadjuvant Radiation"[All Fields] OR "neoadjuvant radiation therap*"[All Fields] OR "neoadjuvant radiation treatment*"[All Fields] OR ("Neoadjuvant Therapy"[MeSH Terms] OR ("neoadjuvant"[All Fields] AND "therapy"[All Fields]) OR "Neoadjuvant Therapy"[All Fields] OR ("neoadjuvant"[All Fields] AND "radiations"[All Fields])) OR "neoadjuvant radiotherap*"[All Fields] OR "Neoadjuvant Systemic Therapies"[All Fields] OR "Neoadjuvant Systemic Therapy"[All Fields] OR "neoadjuvant systemic treatment*"[All Fields] OR "Neoadjuvant Therapies"[All Fields] OR "Neoadjuvant Therapy"[All Fields] OR "neoadjuvant treatment"[All Fields] OR "Neoadjuvant Treatments"[All Fields])) AND ("carcinoma, non small cell lung"[MeSH Terms] OR ("lung non small cell cancer"[Title/Abstract] OR "lung non small cell carcinoma"[Title/Abstract] OR "non oat cell lung cancer"[Title/Abstract] OR "non small cell bronchial cancer"[Title/Abstract] OR "Non Small Cell Lung Cancer"[Title/Abstract] OR "non small cell lung carcinoma*"[Title/Abstract] OR "non small cell pulmonary cancer"[Title/Abstract] OR "non small cell pulmonary carcinoma"[Title/Abstract] OR "non squamous NSCLC"[Title/Abstract] OR "nonsmall cell lung cancer"[Title/Abstract] OR "nonsmall cell lung carcinoma"[Title/Abstract] OR "pulmonary non small cell carcinoma"[Title/Abstract])) AND ("Fluorodeoxyglucose F18"[MeSH Terms] OR "fluorodeoxyglucose F18-maleimidehexyloxime"[Supplementary Concept] OR ("positron emission tomography"[MeSH Terms] OR "Positron Emission Tomography Computed Tomography"[MeSH Terms]) OR ("Single Photon Emission Computed Tomography Computed Tomography"[MeSH Terms] OR "tomography, x ray computed"[MeSH Terms]) OR ("18F FDG"[Title/Abstract] OR "18F Fluorodeoxyglucose"[Title/Abstract] OR "18FDG"[Title/Abstract] OR "2 Fluoro 2 deoxy D glucose"[Title/Abstract] OR "2 fluoro 2 deoxy d glucose f 18"[Title/Abstract] OR "2 Fluoro 2 deoxyglucose"[Title/Abstract] OR "CAT scan"[Title/Abstract] OR "CAT scanning"[Title/Abstract] OR "Cine CT"[Title/Abstract] OR "computed axial tomography"[Title/Abstract] OR "computed tomographic scan"[Title/Abstract] OR "Computed Tomography"[Title/Abstract] OR "computed tomography scan"[Title/Abstract] OR "Computed X Ray Tomography"[Title/Abstract] OR "computer assisted tomography"[Title/Abstract] OR "computer tomography"[Title/Abstract] OR "computerised axial tomography"[Title/Abstract] OR "computerised tomography"[Title/Abstract] OR "computerized axial tomography"[Title/Abstract] OR "computerized tomography"[Title/Abstract] OR "computerized tomography scan"[Title/Abstract] OR "CT PET"[Title/Abstract] OR "ct pet scan*"[Title/Abstract] OR "CT SPECT"[Title/Abstract] OR "ct spect scan*"[Title/Abstract] OR "ct x ray*"[Title/Abstract] OR "Electron Beam Computed Tomography"[Title/Abstract] OR "Electron Beam Tomography"[Title/Abstract] OR "fludeoxyglucose f 18"[Title/Abstract] OR "fludeoxyglucose f18"[Title/Abstract] OR "fluor 18 FDG"[Title/Abstract] OR "Fluorine 18 fluorodeoxyglucose"[Title/Abstract] OR "Fluorodeoxyglucose F 18"[Title/Abstract] OR "Fluorodeoxyglucose F18"[Title/Abstract] OR "p e t"[Title/Abstract] OR "PET CT"[Title/Abstract] OR "pet ct scan*"[Title/Abstract] OR "pet imaging*"[Title/Abstract] OR "pet scan*"[Title/Abstract] OR "positron emission tomographic scan"[Title/Abstract] OR "positron emission tomographic scanning"[Title/Abstract] OR "positron emission tomography"[Title/Abstract] OR "Positron Emission Tomography Computed Tomography"[Title/Abstract] OR "positron emission tomography imaging*"[Title/Abstract] OR "positron tomography"[Title/Abstract] OR "Single Photon Emission Computed Tomography Computed Tomography"[Title/Abstract] OR "SPECT CT"[Title/Abstract] OR "spect ct scan*"[Title/Abstract] OR "Tomodensitometry"[Title/Abstract] OR "Transmission Computed Tomography"[Title/Abstract] OR "x ray cat scan*"[Title/Abstract] OR "X Ray Computed Tomography"[Title/Abstract] OR "X Ray Computer Assisted Tomography"[Title/Abstract] OR "X Ray Computerized Axial Tomography"[Title/Abstract] OR "X Ray Computerized Tomography"[Title/Abstract] OR "x ray ct scan*"[Title/Abstract] OR "Xray Computed Tomography"[Title/Abstract])) | 276 |

**Web of Science**

| - WOS.IC: 1993 to 2024 - WOS.CCR: 1985 to 2024 - WOS.SCI: 1900 to 2024 - WOS.AHCI: 1975 to 2024 - WOS.BHCI: 2005 to 2024 - WOS.BSCI: 2005 to 2024 - WOS.ESCI: 2015 to 2024 - WOS.ISTP: 1990 to 2024 - WOS.SSCI: 1900 to 2024 - WOS.ISSHP: 1990 to 2024 | 1 | TS=((bronchial non small cell cancer) OR (bronchial non small cell carcinoma) OR (lung non small cell cancer) OR (lung non small cell carcinoma) OR (non oat cell lung cancer) OR (non small cell bronchial cancer) OR (Non Small Cell Lung Cancer) OR (non small cell lung carcinoma*) OR (non small cell pulmonary cancer) OR (non small cell pulmonary carcinoma) OR (non squamous NSCLC) OR (nonsmall cell carcinoma of the lung) OR (nonsmall cell lung cancer) OR (nonsmall cell lung carcinoma) OR (pulmonary non small cell cancer) OR (pulmonary non small cell carcinoma)) | Web of Science | 128168 | Sun Sep 29 2024 23:49:27 GMT+0800 |
| --- | --- | --- | --- | --- | --- |
| - WOS.IC: 1993 to 2024 - WOS.CCR: 1985 to 2024 - WOS.SCI: 1900 to 2024 - WOS.AHCI: 1975 to 2024 - WOS.BHCI: 2005 to 2024 - WOS.BSCI: 2005 to 2024 - WOS.ESCI: 2015 to 2024 - WOS.ISTP: 1990 to 2024 - WOS.SSCI: 1900 to 2024 - WOS.ISSHP: 1990 to 2024 | 2 | TS=((neo adjuvant therapy) OR (neo adjuvant treatment) OR (Neoadjuvant Chemoradiation*) OR (Neoadjuvant Chemoradiotherap*) OR (Neoadjuvant Chemotherap*) OR (Neoadjuvant Radiation*) OR (Neoadjuvant Radiotherap*) OR (neoadjuvant therap*) OR (neoadjuvant treatment*)) | Web of Science | 77637 | Mon Sep 30 2024 00:02:20 GMT+0800 |
| - WOS.IC: 1993 to 2024 - WOS.CCR: 1985 to 2024 - WOS.SCI: 1900 to 2024 - WOS.AHCI: 1975 to 2024 - WOS.BHCI: 2005 to 2024 - WOS.BSCI: 2005 to 2024 - WOS.ESCI: 2015 to 2024 - WOS.ISTP: 1990 to 2024 - WOS.SSCI: 1900 to 2024 - WOS.ISSHP: 1990 to 2024 | 3 | TS=((‘18F FDG) OR (18F Fluorodeoxyglucose) OR (18FDG) OR (2 deoxy 2 fluoro d glucose f 18) OR (2 deoxy 2 fluoro dextro glucose f 18) OR (2 deoxy 2 fluoro glucose f 18) OR (2 deoxy 2 fluoroglucose f 18) OR (2 deoxy d glucose f 18) OR (2 deoxy dextro glucose f 18) OR (2 deoxyfluoroglucose f 18) OR (2 deoxyglucose f 18) OR (2 Fluoro 2 deoxy D glucose) OR (2 fluoro 2 deoxy d glucose f 18) OR (2 Fluoro 2 deoxyglucose) OR (2 fluoro 2 deoxyglucose f 18) OR (2 fluorodeoxyglucose f 18) OR (CAT scan*) OR (Cine CT) OR (computed tomographic scan) OR (Computed Tomography) OR (computer tomography) OR (computerised tomography) OR (computerized tomography) OR (CT PET ) OR (CT SPECT*) OR (CT X Ray*) OR (deoxyfluoroglucose f 18) OR (deoxyglucose f 18) OR (Electron Beam Tomography) OR (fludeoxyglucose f 18) OR (fludeoxyglucose f18) OR (fluor 18 FDG) OR (Fluorine 18 fluorodeoxyglucose) OR (fluoro 2 deoxy d glucose f 18) OR (fluoro 2 deoxyglucose f 18) OR (fluorodeoxy d glucose f 18) OR (Fluorodeoxyglucose F 18) OR (Fluorodeoxyglucose F18) OR (glucotrace) OR (meta trace fdg) OR (p.e.t.) OR (PET CT) OR (PET Imaging*) OR (PET Scan*) OR (positron emission tomographic scan*) OR (positron tomography) OR (SPECT CT) OR (steripet) OR (Tomodensitometry) OR (X Ray CAT Scan*) OR (X Ray Computer Assisted Tomography) OR (X Ray CT Scan*')) | Web of Science | 702235 | Mon Sep 30 2024 00:05:16 GMT+0800 |
| - WOS.IC: 1993 to 2024 - WOS.CCR: 1985 to 2024 - WOS.SCI: 1900 to 2024 - WOS.AHCI: 1975 to 2024 - WOS.BHCI: 2005 to 2024 - WOS.BSCI: 2005 to 2024 - WOS.ESCI: 2015 to 2024 - WOS.ISTP: 1990 to 2024 - WOS.SSCI: 1900 to 2024 - WOS.ISSHP: 1990 to 2024 | 4 | #1 AND #2 AND #3 | Web of Science | 363 | Mon Sep 30 2024 00:06:13 GMT+0800 |

**Embase**

| No. | Query | Results | Date |
| --- | --- | --- | --- |
| #12 | #9 AND #10 AND #11 | 1096 | 28-Sep-24 |
| #11 | #5 OR #6 OR #7 OR #8 | 1607556 | 28-Sep-24 |
| #10 | #3 OR #4 | 100619 | 28-Sep-24 |
| #9 | #1 OR #2 | 246457 | 28-Sep-24 |
| #8 | '18f fdg':ti,ab,kw OR '18f fluorodeoxyglucose':ti,ab,kw OR '18fdg':ti,ab,kw OR '2 deoxy 2 fluoro d glucose f 18':ti,ab,kw OR '2 deoxy 2 fluoro dextro glucose f 18':ti,ab,kw OR '2 deoxy 2 fluoro glucose f 18':ti,ab,kw OR '2 deoxy 2 fluoroglucose f 18':ti,ab,kw OR '2 deoxy d glucose f 18':ti,ab,kw OR '2 deoxy dextro glucose f 18':ti,ab,kw OR '2 deoxyfluoroglucose f 18':ti,ab,kw OR '2 deoxyglucose f 18':ti,ab,kw OR '2 fluoro 2 deoxy d glucose':ti,ab,kw OR '2 fluoro 2 deoxy d glucose f 18':ti,ab,kw OR '2 fluoro 2 deoxyglucose':ti,ab,kw OR '2 fluoro 2 deoxyglucose f 18':ti,ab,kw OR '2 fluorodeoxyglucose f 18':ti,ab,kw OR 'cat scan*':ti,ab,kw OR 'cine ct':ti,ab,kw OR 'computed tomographic scan':ti,ab,kw OR 'computed tomography':ti,ab,kw OR 'computer tomography':ti,ab,kw OR 'computerised tomography':ti,ab,kw OR 'computerized tomography':ti,ab,kw OR 'ct pet':ti,ab,kw OR 'ct spect*':ti,ab,kw OR 'ct x ray*':ti,ab,kw OR 'deoxyfluoroglucose f 18':ti,ab,kw OR 'deoxyglucose f 18':ti,ab,kw OR 'electron beam tomography':ti,ab,kw OR 'fludeoxyglucose f 18':ti,ab,kw OR 'fludeoxyglucose f18':ti,ab,kw OR 'fluor 18 fdg':ti,ab,kw OR 'fluorine 18 fluorodeoxyglucose':ti,ab,kw OR 'fluoro 2 deoxy d glucose f 18':ti,ab,kw OR 'fluoro 2 deoxyglucose f 18':ti,ab,kw OR 'fluorodeoxy d glucose f 18':ti,ab,kw OR 'fluorodeoxyglucose f 18':ti,ab,kw OR 'fluorodeoxyglucose f18':ti,ab,kw OR 'glucotrace':ti,ab,kw OR 'meta trace fdg':ti,ab,kw OR 'p.e.t.':ti,ab,kw OR 'pet ct':ti,ab,kw OR 'pet imaging*':ti,ab,kw OR 'pet scan*':ti,ab,kw OR 'positron emission tomographic scan*':ti,ab,kw OR 'positron tomography':ti,ab,kw OR 'spect ct':ti,ab,kw OR 'steripet':ti,ab,kw OR 'tomodensitometry':ti,ab,kw OR 'x ray cat scan*':ti,ab,kw OR 'x ray computer assisted tomography':ti,ab,kw OR 'x ray ct scan*':ti,ab,kw | 630889 | 28-Sep-24 |
| #7 | 'computer assisted tomography'/exp | 1528245 | 28-Sep-24 |
| #6 | 'positron emission tomography'/exp | 254686 | 28-Sep-24 |
| #5 | 'fluorodeoxyglucose f 18'/exp | 83351 | 28-Sep-24 |
| #4 | 'neo adjuvant therapy':ti,ab,kw OR 'neo adjuvant treatment':ti,ab,kw OR 'neoadjuvant chemoradiation*':ti,ab,kw OR 'neoadjuvant chemoradiotherap*':ti,ab,kw OR 'neoadjuvant chemotherap*':ti,ab,kw OR 'neoadjuvant radiation*':ti,ab,kw OR 'neoadjuvant radiotherap*':ti,ab,kw OR 'neoadjuvant therap*':ti,ab,kw OR 'neoadjuvant treatment*':ti,ab,kw | 78807 | 28-Sep-24 |
| #3 | 'neoadjuvant therapy'/exp | 61446 | 28-Sep-24 |
| #2 | 'bronchial non small cell cancer':ti,kw,ab OR 'bronchial non small cell carcinoma':ti,kw,ab OR 'lung non small cell cancer':ti,kw,ab OR 'lung non small cell carcinoma':ti,kw,ab OR 'non oat cell lung cancer':ti,kw,ab OR 'non small cell bronchial cancer':ti,kw,ab OR 'non small cell lung cancer':ti,kw,ab OR 'non small cell lung carcinoma*':ti,kw,ab OR 'non small cell pulmonary cancer':ti,kw,ab OR 'non small cell pulmonary carcinoma':ti,kw,ab OR 'non squamous nsclc':ti,kw,ab OR 'nonsmall cell carcinoma of the lung':ti,kw,ab OR 'nonsmall cell lung cancer':ti,kw,ab OR 'nonsmall cell lung carcinoma':ti,kw,ab OR 'pulmonary non small cell cancer':ti,kw,ab OR 'pulmonary non small cell carcinoma':ti,kw,ab | 145577 | 28-Sep-24 |
| #1 | 'non small cell lung cancer'/exp | 233604 | 28-Sep-24 |

**Cochrane**

| Date Run: | 29/09/2024 12:36:38 |  |
| --- | --- | --- |
| Comment: |  |  |
| ID | Search | Hits |
| #1 | MeSH descriptor: [Carcinoma, Non-Small-Cell Lung] explode all trees | 6701 |
| #2 | ('bronchial non small cell cancer' OR 'bronchial non small cell carcinoma' OR 'lung non small cell cancer' OR 'lung non small cell carcinoma' OR 'non oat cell lung cancer' OR 'non small cell bronchial cancer' OR 'Non Small Cell Lung Cancer' OR 'non small cell lung carcinoma*' OR 'non small cell pulmonary cancer' OR 'non small cell pulmonary carcinoma' OR 'non squamous NSCLC' OR 'nonsmall cell carcinoma of the lung' OR 'nonsmall cell lung cancer' OR 'nonsmall cell lung carcinoma' OR 'pulmonary non small cell cancer' OR 'pulmonary non small cell carcinoma'):ti,kw,ab | 17549 |
| #3 | MeSH descriptor: [Neoadjuvant Therapy] explode all trees | 2672 |
| #4 | (’neo adjuvant therapy' OR 'neo adjuvant treatment' OR 'Neoadjuvant Chemoradiation' OR 'Neoadjuvant Chemoradiation Therap*' OR 'Neoadjuvant Chemoradiation Treatment*' OR 'Neoadjuvant Chemoradiations' OR 'Neoadjuvant Chemoradiotherapies' OR 'Neoadjuvant Chemoradiotherapy' OR 'Neoadjuvant Chemotherapies' OR 'Neoadjuvant Chemotherapy' OR 'Neoadjuvant Chemotherapy Treatment*' OR 'Neoadjuvant Radiation' OR 'Neoadjuvant Radiation Therap*' OR 'Neoadjuvant Radiation Treatment*' OR 'Neoadjuvant Radiations' OR 'Neoadjuvant Radiotherap*' OR 'Neoadjuvant Systemic Therapies' OR 'Neoadjuvant Systemic Therapy' OR 'Neoadjuvant Systemic Treatment*' OR 'Neoadjuvant Therapies' OR 'neoadjuvant therapy' OR 'neoadjuvant treatment' OR 'Neoadjuvant Treatments‘):ti,ab,kw | 13059 |
| #5 | (18F-Fluorodeoxyglucose):ti,ab,kw OR (Positron Emission Tomography):ti,ab,kw OR (Computed Tomography):ti,ab,kw | 26889 |
| #6 | ('18F FDG' OR '18F Fluorodeoxyglucose' OR '18FDG' OR '2 deoxy 2 fluoro d glucose f 18' OR '2 deoxy 2 fluoro dextro glucose f 18' OR '2 deoxy 2 fluoro glucose f 18' OR '2 deoxy 2 fluoroglucose f 18' OR '2 deoxy d glucose f 18' OR '2 deoxy dextro glucose f 18' OR '2 deoxyfluoroglucose f 18' OR '2 deoxyglucose f 18' OR '2 Fluoro 2 deoxy D glucose' OR '2 fluoro 2 deoxy d glucose f 18' OR '2 Fluoro 2 deoxyglucose' OR '2 fluoro 2 deoxyglucose f 18' OR '2 fluorodeoxyglucose f 18' OR 'CAT scan*' OR 'Cine CT' OR 'computed tomographic scan' OR 'Computed Tomography' OR 'computer tomography' OR 'computerised tomography' OR 'computerized tomography' OR 'CT PET ' OR 'CT SPECT*' OR 'CT X Ray*' OR 'deoxyfluoroglucose f 18' OR 'deoxyglucose f 18' OR 'Electron Beam Tomography' OR 'fludeoxyglucose f 18' OR 'fludeoxyglucose f18' OR 'fluor 18 FDG' OR 'Fluorine 18 fluorodeoxyglucose' OR 'fluoro 2 deoxy d glucose f 18' OR 'fluoro 2 deoxyglucose f 18' OR 'fluorodeoxy d glucose f 18' OR 'Fluorodeoxyglucose F 18' OR 'Fluorodeoxyglucose F18' OR 'glucotrace' OR 'meta trace fdg' OR 'p.e.t.' OR 'PET CT' OR 'PET Imaging*' OR 'PET Scan*' OR 'positron emission tomographic scan*' OR 'positron tomography' OR 'SPECT CT' OR 'steripet' OR 'Tomodensitometry' OR 'X Ray CAT Scan*' OR 'X Ray Computer Assisted Tomography' OR 'X Ray CT Scan*'):ti,ab,kw | 37834 |
| #7 | #1 OR #2 | 17549 |
| #8 | #3 OR #4 | 13059 |
| #9 | #5 OR #6 | 37834 |
| #10 | #7 AND #8 AND #9 | 73 |

**Supplementary Table 2:** Demographic and baseline clinical features of the subjects.

| ID | Author | Year | Country | Type of study | Pathological interpretation criteria | N(M/F) | Age（mean±SD） | Neoadjuvant therapy | 18F-FDG PET/CT | SUVmax (Cutoff/Sensitivity/Specificity) | SULpeak (Cutoff/Sensitivity/Specificity) | △SUVmax% (Cutoff/Sensitivity/Specificity) | MTV (Cutoff/Sensitivity/Specificity) | △MTV%(Cutoff/Sensitivity/Specificity) | TLG (Cutoff/Sensitivity/Specificity) | △TLG% (Cutoff/Sensitivity/Specificity) |
| --- | --- | --- | --- | --- | --- | --- | --- | --- | --- | --- | --- | --- | --- | --- | --- | --- |
| 1 | Xiaowei Chen et al | 2023 | China | retrospective study | MPR was defined as less than 10% viable tumor cells in primary tumor bed | 104(92/12) | 63±7.4 | ICI monotherapy (I-M) cohort (36 cases) and ICI combination therapy (I-C) cohort (68 cases) | before and after neoadjuvant ICIs treatment based | MPR:(/91%/77%),(/93%/87%) | NA | MPR:(-35.1%/100%/100%)、(-53%/82.2/96.5) | NA | NA | NA | NA |
| 2 | Rui Guo et al | 2024 | China | retrospective study | MPR defined as 10% or less of viable residual tumour | 51(45/6) | 59.8±8.6 | received 2–3 cycles of intravenous toripalimab (240 mg) or pembrolizumabon(200 mg) IV q 2-3wks added to nab- paclitaxel (100 mg/m2) IV q 2-3wks or pemetrexed (500 mg/m2) IV q 2-3wks plus cisplatin 75mg/m2 IV q 2-3wks | before and after ICRT | NA | MPR:(2.8/76.9/92) | NA | MPR:(1.9/84.6/52) | MPR:(-63.6%/57.7/88 | MPR:(3.0/92.3/52) | MPR:(-90.9%/69.2/92) |
| 3 | Xiuli Tao et al | 2020 | China | prospective study | MPRwas defined as less than 10% viable tumor cells in primary tumor bed | 36(29/7) | 61±5.5 | received 2 doses of sintilimab | before cycles of sintilimab ,4weeks after CRT | NA | MPR:(6.7/92.3/81.8) | NA | MPR:(16.4/69.2/69.6) | MPR:(-33%/92.3/82.6) | MPR:(87.1/76.9/73.9) | MPR:(-60%/100/95.7) |
| 4 | Jang Yoo et al | 2022 | China | retrospective study | PCRwas deﬁned as no residual viable tumor remaining in the posttherapy pathology specimen | 430(309/121) | 61.8 ±9.6 | The neoadjuvant CCRT consisted of chemotherapy and concurrent thoracic radio-therapy(45Gy) | before initialbefore initial treatment and after neoadjuvant CCRT (21days) | CPR:(3.97/74.1/58.8) | NA | CPR:(-56.5%/88.9%/48.7%) | NA | CPR:(-55.4%/68.5/56.6) | NA | CPR:(-86.2%/68.5/69.1) |
|  |  |  |  |  |  |  |  |  |  |  |  |  |  |  |  |  |
| 5 | Andrea L et al | 2017 | America | retrospective study | PERCIST/pCR was defined as absence of any viable tumor cells within the primary lesion/Near-complete pathologic response was defined as microscopic residual disease (≤1% of viable tumor cells) | 44（24/20） | 61.9±8.7 | neoadjuvant concurrent external beam radiation（dose was 60 Gy） and chemotherapy（carboplatin and paclitaxel） | before initiation neoadjuvant threapy,28-42 days after completion of CRT | NA | NA | CPR:(-50%/84.6/51.9) (-75%/53.8%/77.8), MPR:(-50%/84.2/61.9) (-75%/57.9%/90.5%) | NA | NA | NA | NA |
| 6 | Bahce I et al | 2014 | Netherlands | prospective study | Grade 3（CPR）: pathological complete response; Grade 2b(MPR): less than 10% residual vital tumor cells | 23(15/8) | NA | consisted of 3 courses of platinum based doublet chemotherapy and concurrent radiotherapy（46、50Gy) | before and after neoadjuvant therapy (18 days) | NA | NA | MPR:(-55/85%/100%) | NA | NA | NA | NA |
| 7 | Irene A. Burger et al | 2015 | Switzerland | retrospective study | MPR was defined as less than 10% viable tumor cells in primary tumor bed | 44（25、19） | 62±8.7 | Platinum-based neoadjuvant therapy | before receiving neoadjuvant treatment and before undergoing surgery. | MPR:(6.4/85%/58%) | NA | MPR:(-68/69%/81%) | NA | NA | NA | NA |
| 8 | Robert J et al | 2004 | America | retrospective study | CPR was deﬁned as 1% or less of viable tumor cells detected on pathologic review of the entire resected specimens. | 56(31/25) | 63±8.3 | All received carboplatin and paclitaxel | FDG-PET, and chest CT scans both before and after neoadjuvant therapy | NA | NA | CPR:(-60%/100%/95%) ;(-70%/95%/97%);(-80%/90%/100%);(-90%/63%/100%) | NA | NA | NA | NA |
| 9 | Yingpu Cui et al | 2022 | China | retrospective study | MPR was deﬁned as a 10% or less viable residual tumor in the resected specimen. CPR was deﬁned as no viable tumor on all slides of the entire tumor bed. | 30（24/6） | 60±8.1 | toripalimab;carboplatin;pemetrexed;nab-paclitaxel | base-line before treatment;preoperative 18F-FDG PET/CT was performed three weeks after the completion of neoadjuvant treatment | MPR:(4.2/76.9/81.3)，CPR:(4.2/76.9/81.3) | CPR:(3/69.2/93.8),MPR (2.64/100/80) | NA | NA | NA | CPR:(3.92/100/68.8), MPR:(7.77/100/75) | NA |
| 10 | Zhi-Yong Chen et al | 2022 | china | retrospective study | MPR is defined as ≤10% of the viable tumor | 44(37/7) | 59.2±3.5 | nivolumab, pembrolizumab, and camrelizumab | at baseline and within 1 week before surgery | NA | NA | MPR:(-60%/89.3%/62.5%) | NA | NA | NA | NA |
| 11 | You Cheng et al | 2023 | china | retrospective study | CPR was defined as an absence of residual tumor cells in the tumor bed and lymph nodes, and MPR was defined as < 10% residual tumor cells. | 67(57/10) | 57±11.4 | neoadjuvant treatment with nivolumab/pembrolizumab/camrelizumab/tislelizumab/sintilimab | 2 weeks before treatment.;3–4 weeks after the last treatment | NA | NA | MPR:(-75.8%/92.9%/87.2%) | NA | NA | NA | NA |
| 12 | Minglei Yang et al | 2023 | china | retrospective study | CPRwas defined as no viable tumor cell was present in all slides | 185(170/15) | 61.95±8.65 | Pembrolizumab (200 mg) or nivolumab (360 mg) combined with platinum-based chemotherapy | NA | CPR:( /67.9/51.8) | NA | NA | CPR:(/64.3/26.8) | NA | NA | NA |
| 13 | Fenghui Zhuang et al | 2023 | china | retrospective study | MPR was defined as less than 10% viable tumor cells in primary tumor bed | 129(117/12) | 63±2.2 | cohorts A (chemoimmunotherapy) and B (chemotherapy), respectively, while cohort C (chemoimmunotherapy) | prior to (1–14 days before first treatment) or post (2-4 weeks after the last neoadjuvant immunotherapy cycle) neoadjuvant therapy | MPR:A:(3.565/96.6/85) B: (3.565/100/85.2) C:(3.565/96.3/77.3) | NA | MPR:A:(-54.4%/96.6%/85%) B:(-54.4%/100%/89.9%) | NA | NA | NA | NA |
| 14 | Masayuki Tanahashi et al | 2022 | japan | retrospective study | MPR was defined as less than one-third of the cancer tissue | 72(60/12) | 61±7.1 | 2 courses of platinum doublet therapy | before and after ICRT（4 weeks) | NA | NA | MPR:(-60%/72%/76%) | NA | NA | NA | NA |
